# Classification of Dystonia

**DOI:** 10.3390/life12020206

**Published:** 2022-01-29

**Authors:** Lazzaro di Biase, Alessandro Di Santo, Maria Letizia Caminiti, Pasquale Maria Pecoraro, Vincenzo Di Lazzaro

**Affiliations:** 1 Neurology Unit, Campus Bio-Medico University Hospital Foundation, Via Álvaro del Portillo 200, 00128 Rome, Italy; a.disanto@policlinicocampus.it (A.D.S.); m.caminiti@policlinicocampus.it (M.L.C.); p.pecoraro@policlinicocampus.it (P.M.P.); v.dilazzaro@policlinicocampus.it (V.D.L.); 2 Unit of Neurology, Neurophysiology, Neurobiology, Department of Medicine, Università Campus Bio-Medico di Roma, Via Álvaro del Portillo 21, 00128 Rome, Italy; 3 Brain Innovations Lab, Università Campus Bio-Medico di Roma, Via Álvaro del Portillo 21, 00128 Rome, Italy

**Keywords:** dystonia, clinical diagnosis, classification, etiology

## Abstract

Dystonia is a hyperkinetic movement disorder characterized by abnormal movement or posture caused by excessive muscle contraction. Because of its wide clinical spectrum, dystonia is often underdiagnosed or misdiagnosed. In clinical practice, dystonia could often present in association with other movement disorders. An accurate physical examination is essential to describe the correct phenomenology. To help clinicians reaching the proper diagnosis, several classifications of dystonia have been proposed. The current classification consists of axis I, clinical characteristics, and axis II, etiology. Through the application of this classification system, movement disorder specialists could attempt to correctly characterize dystonia and guide patients to the most effective treatment. The aim of this article is to describe the phenomenological spectrum of dystonia, the last approved dystonia classification, and new emerging knowledge.

## 1. Introduction

Dystonia is a hyperkinetic movement disorder characterized by sustained or intermittent muscle contractions causing abnormal movement or posture. Dystonia has distinct clinical features; however, a wide spectrum of phenomenological presentations may be recognized. Dystonia can present in isolation or in combination with other movement disorders like chorea, myoclonus, tremor, and parkinsonism [1]. Dystonia is one of the most underdiagnosed and misdiagnosed movement disorders. The most common misdiagnosis is between [2]: dystonic tremor and essential tremor, parkinsonian tremor, or psychogenic tremor; dystonic jerks and myoclonus; tic-like dystonia and Tourette syndrome. To aid clinicians in reaching the proper diagnosis, several classifications have been proposed over time. The first classification of dystonia was presented in 1976 [3], and during subsequent years it was modified several times [4,5,6]. The last classification was proposed in 2013 and distinguishes two main axes: axis 1, clinical characteristics, and axis 2, etiology [1]. The classification of dystonia in a single patient should be considered as a dynamic process, subject to re-evaluation in the light of the progression of clinical history and new advances in dystonia research.

The aim of the present review is to describe the phenomenological spectrum of dystonia and to discuss the current classification.

## 2. Definition of Dystonia

The last definition of dystonia (proposed by a consensus of the Movement Disorder Society expert members) is articulated in these three sub-definitions [1]:

1. Dystonia is a movement disorder characterized by sustained or intermittent muscle contractions causing abnormal and often repetitive, movements, postures, or both.
2. Dystonic movements are typically patterned, twisting, and may be tremulous.
3. Dystonia is often initiated or worsened by voluntary action and associated with overflow muscle activation.

## 3. Phenomenological Spectrum

Unlike most branches of Neurology, which aim at defining the localization of the disease, movement disorder specialists’ priority is to characterize the phenomenology of the disease. Hence, physical examination plays a role of utmost importance in clinical practice. To minimize mistakes and variability between specialists, a methodological approach should be encouraged. The first step in examining a patient affected by a movement disorder should be to describe the phenomenology of the disease and, in the case of coexistence of multiple phenomenologies, define the prevailing one and the one manifested at the onset. Secondly, the phenomenology identified as prevalent should be categorized as hypokinetic or hyperkinetic. Indeed, movement disorders can be classified into two main categories: hypokinetic and hyperkinetic. Hypokinetic movement disorders can be characterized by loss of voluntary and automatic movements, reduced amplitude of movements, slowness, and rigidity. Hyperkinetic movement disorders are characterized by abnormal, often repetitive, involuntary movements overlapped to normal motor activity. Dystonia is a hyperkinetic movement disorder.

**Figure 1:**
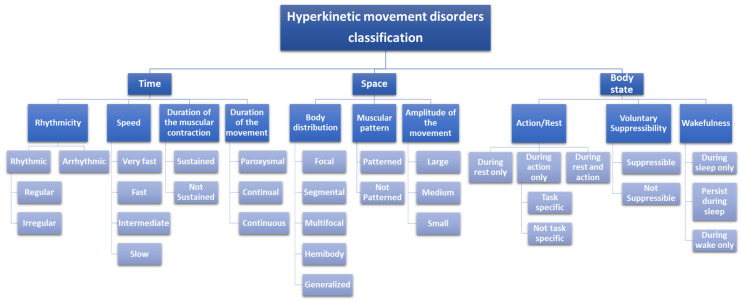
Hyperkinetic movement disorders classification algorithm (modified from [7,8]).

Hyperkinetic movement disorders can be categorized according to different cardinal features [7,8], which describe the movement in terms of (Figure 1): time, space distribution, and body state’s impact.

### 3.1. Time

#### 3.1.1. Rhythmicity

In terms of time, first we describe the rhythmicity [7,8]. A rhythmic movement repeats over time at a fixed interval of time. If the movement can be defined with a frequency during an observation period, it has a regular rhythm (e.g., essential tremor, parkinsonian tremor), if the movement repeats with a more complex temporal pattern, it has an irregular rhythm (e.g., cortical myoclonus), and finally, if the movement repeats over time at no fixed interval of time, it is arrhythmic (e.g., chorea, athetosis, ballism, tics, akathitic movements). Dystonic tremors can have both regular or irregular rhythm, and dystonic movement or posture are generally arrhythmic. Patients affected by dystonia can be misdiagnosed as affected by essential tremor if the patient shows a regular rhythmic kinetic or postural tremor of the arms or of the head, and dystonic posture, which leads to the obfuscation of dystonic tremors during the patient’s examination [2,6]. Another possible misdiagnosis in case of a patient showing a regular rhythmic dystonic tremor at rest is Parkinson’s disease (PD); indeed, it has been supposed that some patients with a clinical diagnosis of PD and without evidence of dopaminergic deficit at DATscan (SWEDD) could be patients affected by dystonic tremor [2,9].

#### 3.1.2. Speed

The second temporal item is the speed ofthe movement [7,8]. It can be very fast, such as in myoclonus or hemifacial spasm, fast las in ballism or tics, intermediate as in chorea and tremors, or slow as in athetosis or akathitic movements. Dystonia can show a large spectrum of speed from very fast in blepharospasm, to fast or intermediate in dystonic tremor, and slow in dystonic postures.

Sometimes dystonia patients can show, during posture, a dystonic spasm [10] of brief duration that can be misdiagnosed as myoclonus [2]. In these cases, EMG recordings could help in differentiating diagnoses since dystonic spasms generally last more than 200 ms [11], which is higher than myoclonus duration. However, true myoclonus can be also present in patients with dystonia as reported for some DYT1 [12,13,14] patients or the combined syndrome myoclonus-dystonia DYT 11-SGCE [15,16].

#### 3.1.3. Duration of the Muscular Contraction

Finally, in terms of the time spectrum, we need to describe the duration of the muscular contraction and the duration of the whole movement [7]. The duration of the muscular contraction can be sustained or not. A sustained muscular contraction is fixed and doesn’t change during time, unlike the non-sustained muscular contraction. According to the current definition, dystonia is a movement disorder characterized by sustained or intermittent muscle contractions [1]. Dystonia can have two components: the tonic component, which leads to dystonic postures, and the phasic component, which leads to dystonic movements, and often these components are both present in the same patient [17,18].

#### 3.1.4. Duration of the Movement

The duration of the movement can be defined as paroxysmal, if the movement repeats with a sudden recurrence (e.g., paroxysmal dyskinesia, paroxysmal ataxia); continual, if the movement repeats over and over again without a sudden recurrence (e.g., ballism, chorea), or continuous, if the movement continues without stopping (e.g., abdominal dyskinesias) [7]. In general dystonia can be, in term of duration of movement, continual in dystonic movements or continuous in dystonic postures. In case of continuous and sustained dystonic posture, the diagnosis is quite simple, but in case of continual dystonic movement, sometimes the differential diagnosis with choreic movement needs more detailed examination, which will show that the dystonic movement has a clear pattern. Instead, the choreic movements are characterized by random muscle contractions. However, dystonia and chorea can be present in the same patient, e.g., in patients affected by Huntington’s disease [2,4].

### 3.2. Space Distribution

#### 3.2.1. Body Distribution

According to space characteristics, we can classify hyperkinetic movements according to body distribution, i.e., the body part involved in the involuntary movement. Dystonia can be classified as [1]:

- Focal: 1 body part is affected
- Segmental: ≥2 contiguous body parts are affected
- Multifocal: ≥2 non-contiguous body parts are affected
- Hemidistonia: Ipsilateral arm and leg are involved
- Generalized: ≥3 body parts are affected, including the trunk and ≥2 other sites; with or without leg involvement

#### 3.2.2. Muscular Pattern

Another important space feature is the muscular pattern activated in dystonia [7]. In patterned movement, the involuntary movements involve the same group of muscles in a repetitive way. According to the current definition of dystonia, dystonic movements are typically patterned [1]; indeed, this feature is one of the most important hallmarks of dystonia that lets us distinguish dystonia from other not patterned, hyperkinetic movement disorders, such as chorea or ballism.

#### 3.2.3. Amplitude

Finally, in terms of space characteristics, it is important to define the amplitude of the movement: large (e.g., ballism), medium (e.g., chorea), or small (e.g., tremor). Generally, in dystonia, the amplitude of the movement can be small in dystonic tremor or medium in dystonic movements.

### 3.3. Body State’s Impact

The last main feature needed to classify a hyperkinetic movement is the impact of body state on the movement. Can the involuntary movement be modified by a voluntary movement? Is it suppressible? Is it modified by wakefulness?

#### 3.3.1. Action Rule

First, we need to evaluate the action rule. Some movements are present only during rest. For example, paradoxical dystonia [19] can be present at rest and disappear during action. Some movements are present during voluntary movement only. In this case, the involuntary movement can be triggered by a general movement of a body part (action dystonia, paroxysmal kinesigenic dystonia) or need a specific task to be triggered (task-specific dystonia) [19]. Other movements are present during both rest and voluntary movement. Some forms of dystonia are present at rest and worse during voluntary movements [19].

According to the definition, dystonia is often initiated or worsened by voluntary action and can be associated with overflow muscle activation [1]. Overflow muscle activation is an involuntary muscle contraction in an anatomic site contiguous to the site involved in the dystonic movement. This generally occurs at the peak of dystonic movements. [1,17,20,21].

Another important supporting diagnostic feature of dystonia is the presence of mirror dystonia. When the affected body side is at rest and a specific task is performed by the unaffected homologous opposite body side, if dystonic movements/postures with the same or similar features of spontaneous dystonic movements/postures are elicited on the affected body side, mirror dystonia feature is present [1,17,21]. Generally, at least three different types of repetitive tasks (e.g., finger sequence, normal writing, or piano-like movements), should be performed, at low and fast speed, in the unaffected limb to determine the presence of mirror dystonia [17].

#### 3.3.2. Suppressibility

Another important feature is suppressibility [7,8]. We need to detect if the movement is totally or partially voluntary suppressible (e.g., stereotypies, tics, akathitic movements), or if it is not suppressible (e.g., myoclonus).

Some dystonic movements are suppressible, and a typical feature of dystonia is the presence of the gestes antagonists (or sensory tricks) [22,23,24,25,26,27].

A sensory trick is a voluntary movement that can alleviate dystonia with a simple touch and without forcefully contrasting the dystonic movement. The effect of the sensory trick is reversible and starts at the same time or soon after the sensory trick movement and disappears when the voluntary movement ends or before its end [17]. The sensory trick is not only a simple sensory phenomenon but is the result of a complex mechanism, including alteration in sensorimotor integration [2].

#### 3.3.3. Wakefulness

Finally, we need to understand the relationship between movement and wakefulness, e.g., if it is present while awake or sleeping [7]. Some movements appear during sleep only (e.g., REM sleep behavior disorder, periodic movements in sleep), or persist during sleep (e.g., spinal myoclonus, myokimia, moving toes) and others are present only while awake. Except for severe dystonia that can persists during sleep, generally, dystonia is only present while awake, like most movement disorders.

Dystonia can manifest as the main phenomenological feature of certain diseases and in combination with other movement disorders, such chorea, myoclonus, tremor, and parkinsonism. The phenomenological spectrum is very important for profiling the movement disorder and reaching the correct diagnosis. It is an induction exercise where we describe all the features of the pathologic movement and at the end, the combination of these features will tell us if the movement can fit with the definition of dystonia or other movement disorders. For example, we can hypothesize two specific clinical cases: a dystonic tremor and a cervical dystonia. If we visit a patient for the first time with dystonic tremor, following the phenomenological spectrum description this movement could be:

- Time: rhythmic; with intermediate speed; associated with non-sustained (intermittent) muscular contraction; with a continual duration (repeats over and over again without a sudden recurrence).
- Space: focal, patterned with a small amplitude.
- Body state: initiated or worsened by voluntary action, not suppressible, and present during wake only.

Instead, a patient with cervical dystonia could have the following phenomenological spectrum:

- Time: arrhythmic; with slow speed; associated with sustained muscular contraction; with a continuous duration (continue without stopping).
- Space: focal, patterned with a small amplitude.
- Body state: present during both rest and action, not suppressible and present during waking time only.

Even if these are two different movements, both fit the definition of dystonia because both are patterned movement associated with a pathological muscular contraction, which is intermittent (phasic) in the case of dystonic tremor and sustained in the case of cervical dystonia, the tremor can be classified as a dystonic movement and the cervical dystonia as a dystonic posture.

## 4. Classifications of Dystonia

Once a movement disorder has been defined as dystonic in nature, further characterization according to current classification should be attempted. Starting with the first classification in 1976 [3], many classifications have followed [4,5,6]. Current classification of dystonia was proposed by a consensus of the Movement Disorder Society expert members in 2013 [1] (Table 1).

The classification is not only an intellectual exercise aimed at diagnosing a patient with the right label, but it is necessary to plan a rational diagnostic approach, defining the prognosis and the right therapy [1].

**Figure 2:**
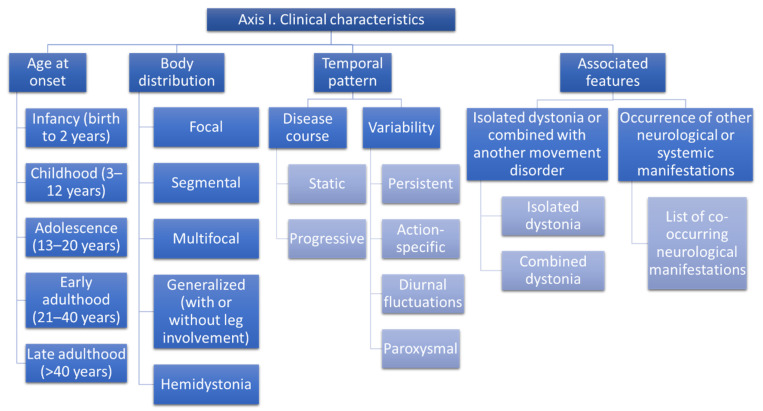
Classification of dystonia. Axis 1 Clinical characteristics [1].

**Figure 3:**
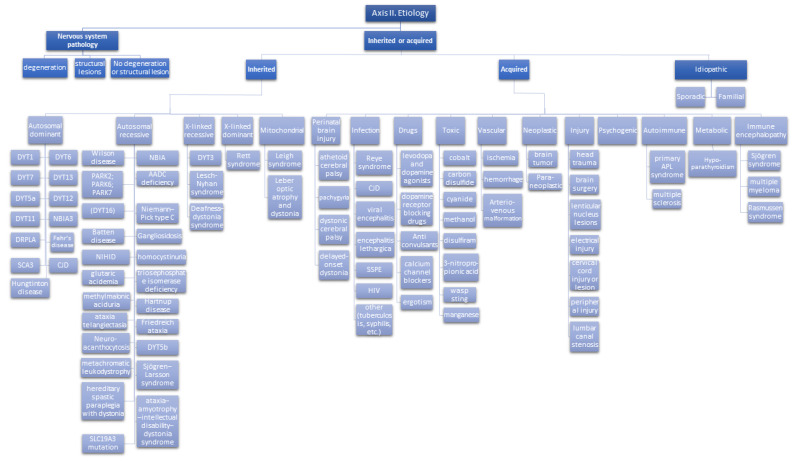
Classification of dystonia. Axis 2 Etiology (modified from [1,7]).

The new classification system proposes an innovative view on how to classify dystonia [1]. The classification distinguishes two main axes:

- Axis 1: Clinical characteristics (Figure 2)
- Axis 2: Etiology (Figure 3)

The two axes are needed since each pattern of clinical characteristics could lead to different etiologies, and therefore there isn’t a unique link between the clinical spectrum and the etiology.

In addition, for a single patient the classification on the 2 axes can be updated over time in a nonparallel way, since the clinical spectrum can evolve during the disease course, but the etiology remains unchanged unless new evidence updates this axis [1].

Axis 1 allows a snapshot of the clinical features of the patient to be taken and to recognize the syndromic pattern. Axis 1 characterization will orient the selection of diagnostic tests to define the etiology (i.e., genetic testing, imaging, lab, or instrumental tests) [1].

### 4.1. Axis I: Clinical Characteristics

#### 4.1.1. Age at Onset

According to age at onset, the new classification includes the following age range [1]:

- Infancy (birth to 2 years)
- Childhood (3–12 years)
- Adolescence (13–20 years)
- Early adulthood (21–40 years)
- Late adulthood (>40 years)

This classification is in line with other neurological disorder classifications and guides the prognosis and the diagnosis definition. For example, dystonia, in infancy, is often due to a metabolic disorder [1,28], and dystonia that emerges in childhood could evolve from focal to generalized, and sporadic types have an onset in adulthood [1].

#### 4.1.2. Body Distribution

Defining the body distribution is important to guide both the prognosis and the therapy. Dystonia can involve different body parts: the head, the neck, the trunk, or the limbs [1]. We have already described the definition for dystonia body distribution in the Phenomenological spectrum/space distribution paragraph. Here we report the classification list:

- Focal
- Segmental
- Multifocal
- Generalized
- Hemidystonia

#### 4.1.3. Temporal Pattern

As described in the phenomenological spectrum, defining the temporal pattern of a hyperkinetic movement disorder is mandatory. The dystonia classification includes two sub-items in temporal pattern: disease course and variability [1].

Disease course:

- Static
- Progressive

Variability:

- Persistent
- Action-specific
- Diurnal fluctuations
- Paroxysmal

The first item is useful to describe the prognosis and the second one to describe when the clinical manifestation occurs.

#### 4.1.4. Associated Features

This item of the classification focuses on the clinical spectrum of movement disorders present in the single patient, and distinguishes the following sub-items [1]:

- Isolated dystonia: dystonia, associated or not with tremors that are the only additional movement disorder
- Combined dystonia: dystonia is combined with other movement disorders
- Complex dystonia: dystonia is accompanied by neurologic or systemic manifestations beyond movement disorders

This classification can guide further testing for etiology identification since dystonia can be a manifestation, for example, of some degenerative parkinsonism, such as progressive supranuclear palsy (PSP) [29] and corticobasal degeneration (CBD) [30,31], and also of some genetic forms characterized by a combination of different movement disorders (Figure 2).

The subdivision into isolated or combined dystonias appeared insufficient to include all the conditions that present dystonia among the clinical manifestations; therefore, a new category has been proposed, that of complex dystonia [32], in which dystonia is accompanied by other neurological (non-movement disorders) or systemic manifestations. A classic example is Wilson disease, characterized by dystonia and other neurological, psychiatric, and liver manifestations [1,33].

This category encompasses conditions in which dystonia dominates the clinical picture in the context of a complex phenotype including symptoms other than movement disorder. Subsequent studies further characterized this new category of dystonia and defined main clinical and genetic syndromes, which fall in this category [32]. The debate around this new category was further enriched using whole exome sequencing in patients with early-onset and familial dystonia, which identified a potential overlap between neurodevelopmental disorder and dystonia [34,35,36]. The main point in this type of patient is to distinguish between dystonia as currently defined and abnormal dystonic postures due to immature or distorted development [37]. Moreover, a recent review highlighted the overlap between heritability of psychiatric disorders and genetic dystonia, probably relying on shared pathophysiological mechanisms [38]. The debate about complex dystonia is still open and the category of complex dystonia will probably be further enriched.

### 4.2. Axis II: Etiology

After the clinical spectrum has been defined with Axis 1, the etiology can be defined with different lab, instrumental, and imaging tests.

Etiology is classified first according to nervous system pathology, with the evidence of degeneration or structural lesions or no evidence of nervous system involvement.

In addition, dystonia is defined according to cause definition: inherited (proven genetic origin), acquired (due to a known non genetic specific cause), or idiopathic (Figure 3) [1].

#### 4.2.1. Inherited Dystonia

Genetic transmission could be distinguished as autosomal dominant, autosomal recessive, X-linked recessive, X-linked dominant, and mitochondrial (Table 2). In this section, the new genetic dystonia nomenclature proposed by MDS Task Force for the Nomenclature of Genetic Movement Disorders is used [39].

#### 4.2.2. Autosomal Dominant

Among autosomal dominant dystonia, several forms of dystonia could be listed: Oppenheim dystonia (DYT-TOR1A), childhood and adult onset-familial cranial limb dystonia (DYT-THAP1), dopa-responsive dystonia (DYT/PARK-GCH1), rapid-onset dystonia–parkinsonism (DYT/PARK-ATP1A3), myoclonus–dystonia (DYT-SGCE), neuroferritinopathy (NBIA/CHOREA-FTL), dentatorubral-pallidoluysian atrophy, Huntington disease, Machado–Joseph disease (SCA-ATXN3), Creutzfeldt–Jakob disease, and Primary Familial Brain Calcification [7].

Oppenheim dystonia (DYT-TOR1A) is caused by a specific 3-base pair deletion “GAG” in the coding region for TorsinA in the TOR1A gene [40]. TOR1A mutation has a reduced penetrance with a variable expressivity ranging from severe childhood-onset generalized to late-onset focal dystonia, and about two thirds of the mutation carriers remain unaffected throughout their life [41]. (DYT-TOR1A) dystonia usually begins in childhood, adolescence, or early adulthood with a mean age of 13 years, a range of 1–28 years with twisting of an arm or leg and progress to involve other limbs and torso, but usually not the face and neck [42]. (DYT-TOR1A) dystonia shows an excellent short-term and long-term response to GPi DBS [43].

Childhood and adult onset familial cranial and limb dystonia (DYT-THAP1) is caused by a mutation in the THAP1 gene on 8p11.21. Clinical phenotype ranges from childhood to adult onset, site of onset could be in arm or cranial (including laryngeal), occasionally leg or neck and usually remains restricted as upper body involvement [7].

Adult-onset familial torticollis (DYT7) is an autosomal dominant form of focal dystonia not replicated since first described in 1996 in a north German family [44]. In the described family, this form of dystonia is mostly of adult onset, occasionally in adolescence, and is limited to neck in 85% of cases [7,44].

Adult-onset familial cervical-cranial predominant dystonia (DYT13) is an autosomal dominant form of adult onset, focal/segmental dystonia, described in non-Jewish families that do not link to DYT1 [45,46], two unrelated consanguineous Brazilian families, [47] and in a German boy [48]. The site of onset is usually in the neck, which continues to dominate, but dystonia often spreads to involve the cranial structures as well, and occasionally the arm [7]. However, the status of the mutation is still unconfirmed as it has not been further replicated [16].

Dopa-responsive dystonia (DYT/PARK-GCH1) is a form of dystonia-parkinsonism responsible for childhood-onset dystonia with response to low doses of oral administration of levodopa [49]. This form of dopa-responsive dystonia is characterized by parkinsonian aspects and diurnal fluctuation of symptoms and was described first in 1979 by Segawa, hence is known also as Segawa disease [50]. Non-motor features including sleep disturbances, mood disorders, and migraine are present in a considerable subset of affected individuals [51].

Rapid-onset dystonia–parkinsonism (DYT/PARK-ATP1A3) is characterized by a rostra-caudal gradient of involvement including bulbar regions, without response to l-dopa therapy [52]. Age of onset is variable, ranging from 4 to 55 years [49].

Myoclonus–dystonia (DYT-SGCE) is inherited in an autosomal dominant manner with maternal imprinting; most affected individuals inherit the pathogenic variant from their fathers, while those inheriting the variants from their mothers remain unaffected throughout their lives. Age of onset is from childhood or adolescence and involves the neck and upper limbs, while involvement of lower limbs is rare. Myoclonic jerks often affect the neck, trunk, and upper limbs, and are transiently ameliorated by alcohol intake [16]. Dystonic symptoms scarcely respond to pharmacological therapies, while GPi stimulation is of great benefit in reducing motor symptoms [53].

Apart from dystonia-myoclonus caused by SGCE, another mutation responsible for myoclonus-dystonia has been identified as a mutation in the KCTD17 gene. The clinical syndrome is characterized by autosomal dominant transmission, with onset of myoclonic jerks of upper limbs in the first or second decades of life. Progressively, the patient develops prominent dystonia of the craniocervical regions and of the trunk or lower limbs [54]. More recently, an atypical presentation with adult-onset of symptoms and phenotypic spectrum characterized by prominent laryngeal dystonia and subsequent upper limb myoclonus was described [55].

Neurodegeneration with brain iron accumulation type 3, also called neuroferritinopathy (NB1A/CHOREA-FTL), typically presents with progressive adult-onset chorea or dystonia affecting one or two limbs, and subtle cognitive deficits. The movement disorder affects additional limbs within 5 to 10 years and becomes more generalized within 20 years. When present, asymmetry remains throughout the course of the disorder. Most individuals develop a characteristic orofacial action-specific dystonia related to speech that leads to dysarthrophonia. Frontalis overactivity and orolingual dyskinesia are common. Cognitive deficits and behavioral issues become major problems with time [56]. Serum ferritin levels are low in many males and postmenopausal females, but within normal limits for premenopausal females. MR brain imaging is abnormal on all affected individuals and one presymptomatic carrier. A gradient echo brain MRI identifies all symptomatic cases [7].

Dentatorubral-pallidoluysian atrophy (DRPLA) is caused by a CAG trinucleotide repeat expansion (≥48 tandem copies) in the Atrophin-1 (ATN1) gene [57]. DRPLA has a median age of onset at 31 years of age with ataxia and cognitive impairment being cardinal features of the disorder. This disease is characterized by a variable combination of clinical manifestations including ataxia, myoclonus, seizures, dementia, and choreoathetotic movements [58]. Cervical dystonia was described as the primary symptom of the affected members in a family with genetically determined DRPLA [59].

Huntington’s disease (HD) is an autosomal dominant, neurodegenerative disorder caused by a trinucleotide (CAG) repeat expansion in the huntingtin (HTT) gene [60]. Chorea is the most widely recognized type of movement disorder associated with HD, although dystonia, tremor, myoclonus, ataxia, and tics are also recognized [61]. In a cohort of HD patients, dystonia was documented in 91% of cases, with dystonia severity being positively correlated with the duration of motor symptoms at time of assessment, disease burden score, and increasing stage of HD. The upper limbs were the most commonly and severely affected body part, with a reduced functional capacity with worsening dystonic symptoms [62].

Machado–Joseph disease (SCA-ATXN3) is the most common autosomal dominant ataxia worldwide [63] and is caused by a trinucleotide (CAG) expansion located in the 10th exon of the ATXN3 gene. Although cerebellar ataxia is the core manifestation of Machado–Joseph disease, the clinical spectrum is heterogeneous and includes pyramidal signs, extrapyramidal signs, peripheral neuropathy, and non-motor manifestations [64]. Movement disorders are common in Machado–Joseph disease, and include parkinsonism, chorea, tremor, myoclonus, and dystonia [64]. Available estimates of dystonia vary between 5.5% and 33% in different series [64]. In Machado–Joseph disease, dystonia could be both generalized dystonia and focal dystonia [65].

Creutzfeldt-Jakob disease (CJD) is a rare neurodegenerative disease that is mainly characterized by rapidly progressive dementia, myoclonus, ataxia, visual disturbances, extrapyramidal and pyramidal involvement, as well as a kinetic mutism [66].

Several movement disorders including myoclonus, dystonia, choreoathetosis, tremor, hemiballismus, atypical parkinsonian syndromes have been described in a significant number of patients with sporadic, familial, or a new variant of CJD (v-CJD) [64]. Dystonia has also been observed, in many cases of sporadic or familial CJD, as an isolated feature or associated with complex movement disorders. Dystonia, as an early symptom in sporadic or familial CJD, is rare, usually unilateral, and has distal distribution [64]. Dystonic posture can be focal, affecting the upper limb or the neck [64], segmental [67], or hemidystonia [68]. Generalized dystonia has been more commonly reported at later stages of the disease and is sometimes preceded by focal or hemidystonia that had progressively worsened [64].

Primary Familial Brain Calcification is characterized by genetic heterogeneity. This term refers to genetically determined calcification, not only of the basal ganglia but also of other brain structures, in the absence of a known metabolic, toxic, infectious, or traumatic etiology [39]. This condition can be associated with various neuropsychiatric symptoms, most frequently movement disorders, such as parkinsonism, dystonia, chorea, ataxia, tremor [39]. Four types of genetically determined brain calcification have been identified: PFBC-SLC20A2, PFBC-PDGFRB, PFBC-PDGFB, and PFBC-XPR1. PFBC-SLC20A2 is characterized by mixed movement disorder, with dystonia, parkinsonism, and cognitive disfunction [64]. PFBC-PDGFRB differs from the previous one only for the predominance of parkinsonism [69]. In PFBC-PDGFRB, dystonia may predominate [64], whereas PFBC-XPR1 is often asymptomatic and clinically heterogeneous [70].

Mutations of the KMT2B gene cause childhood-onset generalized dystonia transmitted in an autosomal dominant manner. The disease usually begins in lower limbs, and later progresses to other regions with variable severity. Moreover, non-motor symptoms are a characteristic of KMT2B dystonia, along with neurodevelopmental disorder, dysmorphic features, and developmental delay [71].

Recently, analysis of whole-exome sequencing data in a cohort of patients allowed Steel et al. to highlight the role of VPS16 mutation in genetic dystonia [72]. This mutation is transmitted in an autosomal dominant manner and belongs to a group of neurologic conditions known as “HOPS-associated neurologic disorders’ (HOPSANDs)”, which are caused by mutations in genes encoding various components of the autophagic/endolysosomal system [73]. Dystonia is associated with mutation in the VPS16 gene, previously known as Dystonia 30 (DYT30), in a childhood-onset dystonia characterized by prominent oromandibular, cervical, bulbar, or upper limb dystonia, followed by slow progression to generalized dystonia. Neurocognitive impairment with mild intellectual disability and psychiatric manifestation can be detected in some groups of patients [72].

We included DYT-33 in the autosomal dominant group of inherited dystonia because the majority of patients carry a heterozygous mutation with incomplete penetrance and variable expressivity in the EIF2AK2 gene; only one case of homozygous mutation was described. This form of genetic dystonia begins in childhood or adolescence, as a focal or generalized dystonia with slow progression to ambulatory difficulties, dysarthria, and dysphagia. However, the clinical phenotype is variable and more complex neurologic disorders with motor delay, lower limb spasticity, mild developmental delay with cognitive impairments, and nonspecific brain imaging abnormalities were described [74]. One case of DYT-33 dystonia responsive to globus pallidus DBS was described [75].

#### 4.2.3. Autosomal Recessive

The list of autosomal recessive forms of inherited dystonia is continuously growing. Among autosomal recessive dystonia, one can distinguish dystonia-parkinsonism syndromes, such as DYT-PRKRA, dopa-responsive dystonia, such as DYT/PARK-TH, dystonia associated with neurodegeneration, and brain iron accumulation (NBIA/DYT-PANK2, NBIA/DYT/PARK-PLA2G6; aceruloplasminemia, fatty acid hydroxylase-associated neurodegeneration). Autosomal recessive dystonia is also associated with numerous metabolic diseases (Wilson disease, glutaric acidemia, homocystinuria, Niemann-Pick type 2, GM1 and GM2 gangliosidosis, etc.).

Wilson disease is a rare autosomal recessive genetic disorder of copper metabolism caused by a mutation in the gene ATP7B on chromosome 13 [64]. Wilson disease is an important cause of early-onset parkinsonism and dystonia [76]. Most common neurological symptoms are a characteristic flapping tremor, dysarthria, psychiatric symptoms, and age of onset is the first and second decade of life [64]. Dystonia is also a common symptom, present in around two-thirds of patients and can evolve from focal to generalized as the disease progresses [77]. Focal forms of dystonia include blepharospasm, cervical dystonia, or risus sardonicus [76].

Neurodegeneration with brain iron accumulation (NBIA) is a group of inherited neurologic disorders characterized by abnormal accumulation of iron in the basal ganglia (most often in the globus pallidus and/or substantia nigra) [78]. The gene for autosomal recessive neurodegeneration with brain iron accumulation type 1 (NBIA/DYT-PANK2, formerly known as Hallervorden–Spatz syndrome) has been identified as pantothenate kinase (PANK2) [64]. Two clinical forms of PANK2, can be described: the “classic/early”, with rapid progression, gait abnormalities at age ~3 years, and the “typical” PANK2, with onset >10 years and a slower progression. Both forms are clinically characterized by dysarthria, progressive dystonia, rigidity, spasticity, hyperreflexia, extensor toe signs. Retinal degeneration is common and may be detected by electroretinogram several years before onset of visual symptoms. Neuropsychiatric symptoms are more frequent in later-onset form [78].

PLA2G6-associated neurodegeneration (NBIA/DYT/PARK-PLA2G6) comprises a continuum of three phenotypes with overlapping clinical and radiologic features: infantile neuroaxonal dystrophy (INAD), atypical neuroaxonal dystrophy (atypical NAD), and PLA2G6 -related dystonia-parkinsonism.

INAD usually begins between ages six months and three years with psychomotor regression or delay, hypotonia, and progressive spastic tetraparesis. Many affected children never learn to walk or lose the ability shortly after attaining it. Strabismus, nystagmus, and optic atrophy are common. Disease progression is rapid, resulting in severe spasticity, progressive cognitive decline, and visual impairment. Many affected children do not survive beyond their first decade. Atypical NAD shows more phenotypic variability than INAD. In general, onset is in early childhood but can be as late as the end of the second decade. The presenting signs may be gait instability, ataxia, or speech delay and autistic features, which are sometimes the only evidence of disease for a year or more. Strabismus, nystagmus, and optic atrophy are common. Neuropsychiatric disturbances including impulsivity, poor attention span, hyperactivity, and emotional lability are also common. The course is stable during early childhood and resembles static encephalopathy but is followed by neurologic deterioration between ages seven and 12 years [78].

PLA2G6-related dystonia-parkinsonism has a variable age of onset, but most individuals present in early adulthood with gait disturbance or neuropsychiatric changes. Affected individuals consistently develop dystonia and parkinsonism (which may be accompanied by rapid cognitive decline) in their late teens and into their early twenties. Dystonia is most common in the hands and feet but may be more generalized. The most common features of parkinsonism in these individuals are bradykinesia, resting tremor, rigidity, and postural instability [78].

Aceruloplasminemia (NBIA/DYT/PARK-C) is a disorder of iron metabolism caused by the complete absence of ceruloplasmin ferroxidase activity, is associated with very low to absent serum ceruloplasmin and some combination of the following: low serum copper concentration, low serum iron concentration, high serum ferritin concentration, and increased hepatic iron concentration [79]. Aceruloplasminemia (NBIA/DYT/PARK-C) is characterized by iron accumulation in the brain and viscera. The clinical triad of retinal degeneration, diabetes mellitus (DM), and neurologic disease is seen in individuals ranging from age 30 years to older than 70 years. The neurologic findings of movement disorders (blepharospasm, grimacing, facial and neck dystonia, tremors, chorea) and ataxia (gait ataxia, dysarthria) correspond to regions of iron deposition in the brain [79].

Fatty acid hydroxylase-associated neurodegeneration (FAHN) (HSP/NBIA-FA2H) is characterized early in the disease course by central nervous system involvement including corticospinal tract involvement (spasticity), mixed movement disorder (ataxia/dystonia), and eye findings (optic atrophy, oculomotor abnormalities), and, later in the disease course, by progressive intellectual impairment and seizures. With disease progression, dystonia and spasticity compromise the ability to ambulate, leading to wheelchair dependence. Life expectancy is variable. FAHN is a subtype of neurodegeneration with brain iron accumulation (NBIA) [78].

The PARK-Parkin monogenic form of PD is characterized by young or very young age at onset, a good and lasting effect of levodopa, and a lower risk for non-motor symptoms, such as cognitive decline and dysautonomia [80]. Lower limb dystonia, along with rigidity, hyperreflexia, and psychiatric symptoms have been described [79]. Another form of early-onset PD with dystonia is caused by homozygous mutations in phosphatase and tensin homolog-induced putative kinase1 (PARK-PINK1) [81].

DYT/PARK-TH dystonia is a form of partially dopa-responsive infancy-onset dystonia caused by tyrosine hydroxylase deficiency [49]. This form of dystonia is much more severe than dopa-responsive dystonia caused by mutation in GCH1 gene, inherited in an autosomal dominant fashion [82]. DYT/PARK-TH dystonia is characterized by diurnal fluctuation of symptoms, bradykinesia, hypotonia, autonomic disturbances, ptosis, and oculogyric crises [49].

DYT-PRKRA is an autosomal recessive gene inherited form of dystonia, characterized by oromandibular involvement, dysphagia, and retrocollis [49]. Parkinsonian features are mild (or even absent) and do not respond to levodopa therapy [47].

Aromatic-L-amino acid decarboxylase (DYT-DDC) deficiency is a rare autosomal recessive genetic disorder with clinical impacts principally attributable to its effects on neurotransmitter synthesis [83]. The most common symptoms of aromatic-L-amino acid deficiency, each of which was reported in ≥65% of confirmed patients, were hypotonia (typically axial; may be accompanied by limb hypertonia), movement disorders (most frequently oculogyric crises and/or dystonia), development delay, and autonomic symptoms (typically ptosis and/or excessive sweating) [83].

Niemann-Pick type C (NP-C) is a rare autosomal recessive neurodegenerative disorder with an estimated incidence of 1 per 120.000 live births [84]. The disorder is caused by mutations in the NP-C 1 (95%) or NP-C 2 gene (5%). Although the exact cellular functions of the proteins remain to be elucidated, they are involved in cellular lipid transport/trafficking and mutated proteins lead to (lysosomal) accumulation of lipids. Lipid storage products accumulate in many organs, including the brain, resulting in cerebral degeneration with progressive neurological symptoms, cognitive decline, and psychiatric symptoms. NP-C is a very heterogeneous disorder regarding age of onset and clinical presentation. The age of onset can vary from the newborn period to adulthood [85].

The neuronal ceroid lipofuscinoses (NCLs) represent a heterogeneous group of genetically determined neurodegenerative conditions that are characterized by a progressive decline of cognitive and motor capacities, retinopathy evolving into blindness, variable cerebellar atrophy, and myoclonic epilepsy, leading to significantly decreased life expectancy [86]. Recently, a case of status dystonicus associated with CLN8 disease was described [87].

GM1 gangliosidosis is a rare inborn error of metabolism caused by mutations in the galactosidase beta 1 (GLB1) gene leading to a deficiency in the lysosomal enzyme b-galactosidase. This enzyme is involved in the degradation of glycoproteins, glycolipids, and keratan sulfate [88]. GM1 gangliosidosis type III, chronic/adult form (DYT/PARK-GLB1) is characterized by dystonia, parkinsonism, and additional clinical features: pyramidal signs, dysarthria, cognitive deficits (often mild initially), skeletal abnormalities and short statue, corneal clouding, vacuolated cells, cardiomyopathy, and progressive disease [79].

The spectrum of phenotypes resulting from storage of GM2 ganglioside caused by 6-hexosaminidase deficiencies is now quite large. Late infantile or juvenile GM2 gangliosidosis has its onset between 1 and 9 years of age and shows variable features including ataxia, seizures, myoclonus, and, less frequently, cherry-red spots. Dementia is always present [89]. Unsteadiness of gait occurs early in the disease, and later, dysarthria and dementia. The clinical picture is dominated by dystonia and choreiform movements; cerebellar ataxia or spinocerebellar degeneration has been reported as the presenting feature in 23 patients with late-onset GM2 gangliosidosis [79].

Metachromatic leukodystrophy is an inherited lysosomal disorder caused by recessive mutations in ARSA encoding arylsulfatase A. Low activity of arylsulfatase A results in the accumulation of sulfatides in the central and peripheral nervous system, leading to demyelination. The disease is classified in a late-infantile, juvenile, and adult-onset type based on the age of onset, all characterized by a variety of neurological symptoms, which eventually lead to death if untreated [90].

Several inherited defects of sulfur amino acid metabolism may result in homocystinuria (HCU). The most common is a recessively inherited deficiency of the enzyme cystathionine beta-synthase leading to impaired transformation of homocysteine to cystathionine and elevated levels of plasma homocysteine and methionine [91]. Neurological involvement may occur in the form of cerebral thrombotic episodes, seizures, mental retardation, and dystonia [79].

The most common among the organic acidurias, glutaric aciduria type 1 (DYT/CHOR-GCDH), often presents with prominent movement disorders, such as dystonia, parkinsonism, and chorea [76]. GA-1 is caused by autosomal-recessive mutations in glutaryl CoA dehydrogenase [92], leading to a deficiency in this enzyme and subsequent accumulation of neuro- toxic metabolites, 3-hydroxyglutaric acid and glutaric acid [93].

Isolated methylmalonic aciduria (DYT/CHOR-MUT) is an autosomal-recessive disorder of amino acid metabolism caused by impaired activity of the methylmalonyl-coenzyme A mutase enzyme [94]. Although it is primarily a pediatric disorder, undiagnosed cases may present to adult neurologists. Movement disorders (dystonia, chorea, myoclonus, and tremor) occur in 30% to 45% of cases [94].

Hartnup disease (HD) is an autosomal recessive condition characterized by a defect in renal and intestinal membrane transport of mono amino-monocarboxylic acids [95]. Decreased renal tubular reabsorption results in an increase in urine concentration of these amino acids and a characteristic urine amino acid chromatographic pattern, which provides the only accurate diagnostic test for HD. As a result of both decreased intestinal absorption and renal tubular reabsorption, the serum concentration of many amino acids may be decreased [96]. It appears that HD is mainly an asymptomatic disorder unless there is inadequate nutrition in the patient. The usual clinical manifestations of rare symptomatic HD are intermittent, pellagra-like skin rashes, reversible attacks of cerebellar ataxia, and occasionally behavioral changes, ranging from emotional instability to psychosis and delirium. Some patients are mildly retarded. A case of an HD-presenting patient with intermittent dystonia has been described [96].

Ataxia–telangiectasia (A–T) is a clinically heterogeneous disorder, with autosomal recessive inheritance caused by mutations in the A–T mutated (ATM) gene [97]. Even though the typical phenotype of A–T is characterized by cerebellar ataxia, oculocutaneous telangiectasia, and oculomotor apraxia during early childhood, various atypical phenotypes, including later age of onset, absence of ataxia or telangiectasia, and higher survival rate, were also reported [98]. Additionally, besides cerebellar ataxia, various extrapyramidal signs could commonly manifest in A–T patients even without typical characteristics [99]. In particular, dystonia is the second most frequent initial symptom other than ataxia, and the second most prevalent movement disorder following myoclonus during the overall disease course [97].

Friedreich ataxia (FRDA) is the most common of the hereditary ataxias. It is due to GAA triplet repeat expansion in the intronic portion of the frataxin gene [100]. Although a few patients have been found to have point mutations in the frataxin gene [94], the majority have been found to be homozygous for the unstable GAA expansion. With an average age of onset of 10–15 years, FRDA causes progressive ataxia of limbs and gait, dysarthria, cardiomyopathy and an increased rate of diabetes mellitus. Dystonia had been described as a rare feature of FRDA, both focal and segmental [94].

Neuroacanthocytosis is a rare group of neurodegenerative disorders associated with widespread, non-specific nervous system symptoms or acanthocytosis [101]. Neuroacanthocytotis encompasses chorea-acanthocytosis, McLeod syndrome, pantothenate kinase-associated neurodegeneration, and Huntington’s disease-like 2 [102]. The core symptom is basal ganglia degeneration, especially ataxia caused by striatum degeneration [101]. Dystonia is also a common feature, particularly in McLeod syndrome and Huntington’s disease-like 2.

Neuronal intranuclear inclusion disease (NIID), also known as neuronal intranuclear hyaline inclusion disease (NIHID), is a very rare neurodegenerative disorder characterized by the presence of eosinophilic intranuclear inclusions in neuronal and glial cells [103]. Among the numerous neurologic manifestations, craniocervical dystonia [104], axial dystonia [105], lower limb dystonia [106], and dopa-responsive dystonia have been reported [103].

Hereditary spastic paraplegias (HSPs) are rare neurologic disorders that are genetically and clinically heterogeneous [107]. Dystonia has been described in spastic paraplegia type 7 (HSP-SPG7), an autosomal recessive type of HSP that clinically encompasses pure and complex forms. Two patients with cervical dystonia have been described and also a case of a patient who presented with limb dystonia and was found to have HSP associated with a rare compound heterozygous SPG7 mutation [107], dysphagia [108], myopathy and amyotrophy [109], optic neuropathy or atrophy [110], sensory changes [109], vestibular dysfunction [111], and intellectual disability [112].

Sjogren–Larsson syndrome (HSP-ALDH3A2) (SLS) is a rare autosomal recessive neurocutaneous disorder characterized by ichthyosis, spastic di- or tetraplegia and mental retardation, caused by an enzymatic defect in fatty alcohol oxidation [113]. A case of generalized dystonia in SLS was described [114]. Biotin-responsive basal ganglia disease (DYT-SLC19A3) is a recessive disorder caused by mutations of the SLC19A3 gene, coding for a transporter related to the reduced-folate and thiamin [115]. Clinical manifestations are characterized as subacute to acute encephalopathy, sudden loss of developmental milestones, inability to swallow, loss of speech (or slurred speech), loss of motor function, with development of quadriparesis or quadriplegia, and seizures. Left untreated, the disorder results in a chronic or slowly progressive encephalopathy, with an akinetic mute state, permanent loss of speech and comprehension, and eventual death. Dystonia and progressive cogwheel rigidity are accompanying features [115].

In the nomenclature of genetic movement disorders, some genetically determined dystonias waited for independent confirmation [39]. One of these was the DYT 2, a form of childhood-adolescence onset dystonia. Atasu et al. confirmed the mutation in the HPCA (hippocalcine) gene, transmitted in an autosomal recessive manner, as the genetic cause of the DYT2 dystonic syndrome. The phenotypic characterization is variable and includes childhood-onset of generalized dystonia, adolescence-onset of segmental dystonia and a more severe phenotype in which generalized dystonia is accompanied by febrile seizures, dysarthria, and learning difficulties [116].

A novel form of autosomal, recessive-inherited dystonia is caused by mutation in the AOEP gene and corresponds to the Dystonia 31 syndrome. It is a generalized dystonia with a variable onset of symptoms, from 9 to 36 years, with prominent involvement of the upper limbs. The disease course is progressive and speech articulation difficulties, muscle cramping and pain, orofacial dyskinesia, and swallowing dysfunction were described. In a French family, late onset parkinsonism with prominent bradikynesia, dysarthria, and frequent falls was described [117].

#### 4.2.4. X-Linked Recessive

Dystonia-parkinsonism or Lubag sydnrome (DYT/PARK-TAF1) is the only known X-linked form of isolated or combined dystonia where neurodegeneration is documented [49]. DYT/PARK-TAF1 is endemic in the Philippines where is known as “lubag” in the local Filipino dialect, meaning “twisted” [118]. Penetrance is complete in men and almost all women heterozygotes are unaffected. This form of dystonia manifests in the early-late adulthood, involves neck and oromandibular areas, and then progress to generalized with parkinsonian characteristics [119]. Lesch Nyhan syndrome (DYT/CHOR-HPRT) is an inborn disorder caused by a deficiency of the hypoxanthine-guanine phosphoribosyltransferase (HPRT) enzyme, involved in the purine salvage pathway whose inactivation causes an increase in guanine and hypoxanthine, which eventually gets converted into uric acid [120]. The characteristics defining the disease are hyperuricemia, neurodevelopmental abnormalities with global developmental delay, involuntary movements, and self-injurious behavior [121]. Dystonia is the most common extrapyramidal sign and in almost all cases progresses to generalized with patients totally dependent and wheelchair bound [120].

Mohr-Tranebjaerg syndrome (DYT-TIMM8A) is an X-linked recessive syndrome caused by mutations in the nuclear gene TIMM8A, encoding for a protein involved in mitochondrial transport [122]. Main clinical features are sensory-neural deafness and dystonia, whereas other less common features are pyramidal signs, optic atrophy, psychiatric disturbances, and cognitive decline [122,123].

#### 4.2.5. X-Linked Dominant

Rett syndrome is a neurodevelopmental disorder caused in the vast majority of cases by a de novo mutations in the MECP2 gene (encoding methyl-CpG-binding Protein) [124] [125]. Hand stereotypies are core features and other hyperkinetic movement disorders have variously been described in Rett syndrome, including tremor, dystonia, chorea, and myoclonus. For some affected individuals, an evolution from a hyperkinetic to hypokinetic state has also been observed [124].

#### 4.2.6. Mitochondrial

Leigh syndrome, or subacute necrotizing encephalopathy, is an inherited mitochondrial dysfunction accompanied by bilateral basal ganglia lesions, caused by more than 75 monogenic causes [126]. Dystonia is one of the presenting symptoms, along with developmental delay, hypotonia, ataxia, and optical atrophy [127].

Leber’s hereditary ocular neuropathy (LHON) (DYT-mt-ND6) is due to mutations in the mtDNA genes that encode subunits of NADH dehydrogenase and three major point mutations have been described (m.3460G > A, m.11778G > A and m.14484T > C) [128]. In the LHON plus dystonia syndrome, dystonia can precede ocular abnormalities by several years [127]. Dystonia could be generalized and is accompanied by visual loss, pyramidal tract signs, and intellectual impairment [125].

#### 4.2.7. Acquired Forms of Dystonia

In addition to idiopathic and genetic dystonia, different environmental insults that result in brain or spinal or peripheral nervous system damage cause the so-called “acquired dystonia” [7], which is not an inherited disease due to a known specific process including vascular, infection, immunologic, neoplastic, drugs, or toxins (Table 3).

Athetosis cerebral palsy (or dyskinetic cerebral palsy) is the second most-common form of cerebral palsy (CP), that is, a clinical condition characterized by delayed motor development, paresis, and movement disorders (including dystonia). CP is associated with other clinical features like mental retardation and epilepsy and arises after perinatal brain damage [129]. Basal ganglia and thalamus are high-energy metabolism structures and hence are extremely susceptible to hypoxia or ischemia, which are two common causes of cerebral palsy [130]. Dyskinetic cerebral palsy is defined as abnormal posture and movement, including dystonia (dystonic cerebral palsy-DCP). DCP-typical features are delayed onset after perinatal damage, dystonia induced by voluntary movement, association with non-motor symptom, suggestive medical history, and correlation to brain imaging abnormalities [129]. Furthermore, delayed onset dystonia can also occur with other processes like central pontine myelinolysis, cyanide intoxication, electrical injuries, and head trauma [7].

As briefly mentioned before, dystonia can occur if a focal brain lesion affected the basal ganglia (mostly putamen), thalamus, parietal cortex, and cerebellum [129]. The basal ganglia lesions predominantly result in clinical contralateral hemidystonia and the most common etiologies are stroke, trauma, and perinatal injuries [131]. However, it is known that lesion spinal and peripherical lesions can also result in dystonia. Spinal cord lesions (including syringomyelia) and neck trauma can cause cervical dystonia [132]. Peripheral nerve lesions can cause focal delayed-onset dystonia with fixed dystonic posture in the same limb [129].

Several drugs can induce acquired forms of dystonia. The larger part of drug-induced dystonia is produced by dopamine receptor blocking drugs (DRBD) [133]. In particular, D2 receptor blocker drugs are the most involved in the acquired dystonia pathogenesis [134]. Other drugs, like anticonvulsants and calcium channel blockers, can induce acquired dystonia, but incidence is low [129]. Tardive dystonia (TD) is the most frequent, clinical, drug-induced dystonia that start days or year after DRBD exposition 2. Usually, it is characterized by cranial muscle involvement (jaw, tongue, facial muscles) and/or retrocollis. Moreover, TD is often associated with tardive dyskinesia, such as repetitive oral and lingual movement [133]. While acute dystonic reaction and oculogyric crisis usually occur in the first five days after initiation or increase in DRBD therapy including neuroleptics or peripheral DRBD, such as metoclopramide [135]. Conversely, use of dopamine and dopamine agonists can cause hyperkinetic disorders (such as dystonia and dyskinesia) only in Parkinson’s disease patient [7,129].

Toxin and chemical agents can also result in secondary dystonia. Manganese, cyanide and methanol can accumulate in basal ganglia (mainly putamen) and produce focal dystonia [136]. Moreover, metabolic disorder can induce diffuse brain damage that can produce dystonia. Hypoglycemia is a rare cause of dystonia, hypoparathyroidism and consequent hypercalcemia can induce basal ganglia calcification that result in dystonia and finally, hyper or hyponatremia can induce central pontine myelinolysis that results dystonia [137].

Moreover, focal and generalized dystonia can occur as a clinical manifestation of several infection and encephalitis like human immunodeficiency virus (HIV) infection, Creutzfeldt-Jakob disease and progressive multifocal leukoencephalopathy [138]. Immune-mediated disease and autoimmune disorders have been associated with acquired hemidystonia, like primary antiphospholipid syndrome and Sjogren syndrome [139].

Finally, it is still controversial, but to date psychogenic (functional) dystonia are considered an acquired causes of dystonia [1]. This disorder can closely mimic organic dystonia, but some clues can help clinician to differentiate the two disorders: history of psychiatric disorders, abrupt onset, inconsistent and non-patterned movements that changes body regions or muscles involved, incongruent movement, attenuation or disappear with distraction maneuver, false weakness or other atypical features that are not congruent with primary disorder [140].

## 5. Discussion

After the first description by Oppenheim [141], the definition and the classification of dystonia have been updated several times and probably will be further updated in the light of the discovery of more underling etiologies.

For the diagnosis of dystonia, the examiner should follow the definition of dystonia approved in the last expert consensus [1], and focus on the classic five physical signs of dystonia: 2 main physical signs (dystonic movements and dystonic posture) and 3 additional physical signs (mirror dystonia, overflow dystonia, and gestes antagonists/sensory tricks) [17,142].

Overall dystonia diagnosis and classification is challenging. Dystonia has characteristic clinical features, but a wide spectrum of phenomenological presentations. To date, the diagnosis relies mainly on clinical evaluation, and there are no objective biomarkers that can confirm the diagnosis or monitor the evolution of the symptoms.

Since the last proposed classification of dystonia [1], much progress has been made in the field of dystonia. First, the use of whole-exome sequencing made it possible to identify the genetic causes underlying the forms of dystonia whose cause was still unknown. Second, increasing knowledge about the etiology of dystonia lead to the definition of a new category known as complex dystonia along with the classic isolated and combined dystonia categories. In this category, dystonia represents the main neurological disorder in the context of a complex phenotype that includes symptoms other than movement disorders. Furthermore, the debate on complex dystonia has been enriched thanks to the discovery of important overlaps between dystonia and neurodevelopmental disorder and dystonia and psychiatric disorder, both from a genetical and pathophysiological point of view.

It is of paramount importance to recognize and properly characterize patients affected by dystonia to provide them with the most appropriate treatment. Available treatments for dystonia are symptomatic and can improve patients’ quality of life. The most used and well-tolerated treatment for dystonia is the botulin neurotoxin, which is a treatment that can be tailored according to patient symptoms and severity and therefore can be applied to different types of dystonia [143], such as blepharospasm [144,145,146], cervical dystonia [145,147], or task-specific dystonia [148,149,150], limb dystonia [151,152], and trunk dystonia [153,154]. In the last few decades, Deep Brain Stimulation (DBS), has been used in clinics as a therapeutic tool for the treatment of different movement disorders like Parkinson’s disease [155,156,157,158], tremors [159,160,161], and hyperkinetic disorders [162], including dystonia [163]. For intractable (drug refractory) primary dystonia, including generalized and/or segmental dystonia, hemidystonia, and cervical dystonia (torticollis), Deep Brain Stimulation (DBS) in the globus pallidus pars interna (GPi) [163,164,165,166] is an approved treatment. The study of oscillatory activities in neurological disorders [167,168] revealed new pathological biomarkers in recent years. Several authors suggested that these abnormalities could be used as a biomarkers to deliver electrical DBS only in response to pathological neuronal oscillation (adaptative DBS -aDBS); this technique was mainly tested in Parkinson’s disease patients [169,170,171] and it has been suggested that it could be translated to dystonic patients with specific biomarkers, e.g., GPi LFPs theta-alpha band activity [172].

In dystonia, the clinical evaluation, which should be made by an expert in movement disorders, should remain the core of the diagnostic approach, but in the era of big data and artificial intelligence, the use of these new tools to improve the diagnostic accuracy and to accelerate the diagnostic process could be a great opportunity. In the coming years, the approach to patient evaluation and data collection should change and follow an open data approach for the field of movement disorders.

The clinical evaluation can be systematized and videotaped, the movements can be recorded through wearable sensors, and neurophysiological, imaging, lab, and genetic testing can be performed. All these data along with clinical history information can be shared on a platform into a framework of movement disorder experts. To date, there are already a few examples of shared datasets, but the main limitation to their usage is the data entry problem, which is a time-consuming activity that needs dedicated personnel, and it is mainly used for research but not in routine clinical practice.

This is the point that will be a game changer. In order to avoid the data entry problem of limiting the sharing of patient data, the whole dataset should accept three kinds of data: (1) structured data by filling all the fields included into the dataset; (2) structured data coming from a research project that, for data analysis purposes, should be collected in a structured way in any case, following the structure of the local protocol, without modification in order to avoid further data entry work; (3) and unstructured raw data coming from the routine clinical practice patient evaluation.

Following this open structure that allows the collection of both structured and unstructured data, almost all the movement disorder experts that manage data on dystonia patients could collaborate by sharing the platform according to their time availability. Research groups that have dedicated personnel for data entry could provide structured data by filling all the fields included in the dataset; all other collaborators that have no additional time for data entry could just upload and share their patient’s data in the actual format without the need of data entry or new data collection. Potentially, by following this new approach in the near future, we could have the biggest dataset on dystonia ever seen in science.

This new approach that also allows the collection of unstructured data was not possible until a few years ago, but to date it is made possible thanks to modern natural language processing technologies, which transform raw and unstructured data into an exportable and analyzable structured dataset automatically.

The setup of this kind of platform will be an investment for the future, and thanks to new data analysis approaches, such as machine learning and deep learning, the possible future advantages would be in terms of disease characterization, subtype clustering, diagnosis prediction, disease evolution prediction, epidemiological studies, and therapy targeting. However, in parallel with this activity that will give benefits in a medium/long-term period, the classic research should continue with clinical trials aiming to validate objective biomarkers useful for the differential diagnosis of dystonia, for symptoms evolution monitoring, and for therapy efficacy evaluation; this approach can provide a benefit in a relative short-term period.

## 6. Conclusions

Waiting for more advanced and objective tools, the core of diagnosis and classification of dystonia remains an accurate physical examination, which is essential to describe the correct phenomenology. For etiology definition, in selected cases according to the patient’s history and clinical manifestations, genetics is an essential tool for classification and could allow a better understanding of the pathophysiology of dystonia. The field of dystonia is rapidly progressing in both diagnosis and therapy. On the diagnostic side, the classification of dystonia is periodically updated following emerging knowledge. The present review wants to bring attention to dystonia by describing a revision of the classification that encompasses recent discoveries in this field.

## Figures and Tables

**Table 1:** Classifications of dystonia.

Year	Reference
1976	(Fahn and Eldridge) [3]
1987	(Fahn, Marsden et al.) [4]
1998	(Fahn, Marsden et al.) [5]
2011	(Albanese, Asmus et al.) [6]
2013	(Albanese, Bhatia et al.) [1]

**Table 2:** Inherited causes of dystonia.

**Autosomal Dominant**	
**Disease**	**OMIM Code**
- Oppenheim dystonia (DYT-TOR1A)	#128100
- Childhood and adult onset-familial cranial limb dystonia (DYT-THAP1)	#602629
- Dopa-responsive dystonia (DYT/PARK-GCH1)	#128230
- Rapid-onset dystonia–parkinsonism (DYT/PARK-ATP1A3)	#128235
- Myoclonus–dystonia (DYT-SGCE)	#159900
- Neuroferritinopathy (NBIA/CHOREA-FTL)	#606159
- Dentatorubral-pallidoluysian atrophy	#125370
- Huntington’s disease	#143100
- Machado–Joseph disease (SCA-ATXN3)	#109150
- Creutzfeldt–Jakob disease	#123400
- Primary Familial Brain Calcification	#213600
- Myclonic-dystonia 26 (DYT-26)	#616398
- Dystonia-28 (DYT-KMT2B)	#617284
- Dystonia-30 (DYT-30)	#619291
- Dystonia-33 (DYT-33)	#619687
**Autosomal recessive:**	
- Wilson disease	#277900
- Neurodegeneration with brain iron accumulation type 1 (NBIA/DYT-PANK2)	#234200
- Neurodegeneration with brain iron accumulation type 2, infantile neuroaxonal dystrophy (NBIA/DYT/PARK-PLA2G6)	#610217
- Aceruloplasminemia (NBIA/DYT/PARK-C)	#604290
- Fatty acid hydroxylase-associated neurodegeneration (FAHN) (HSP/NBIA-FA2H)	#612319
- Early-onset parkinsonism (PARK-Parkin) (PARK-PINK1)	#608309
- Aromatic-L-amino acid decarboxylase (DYT-DDC)	#608643
- Early-onset dystonia with parkinsonism (DYT-PRKRA)	#612067
- Niemann–Pick type C	#257220
- Juvenile neuronal ceroid-lipofuscinosis (Batten disease)	#204200
- GM1 gangliosidosis (DYT/PARK-GLB1) type III, chronic/adult form	#230500
- GM2 gangliosidosis	#272750
- Metachromatic leukodystrophy	#250100
- Homocystinuria	#277400
- Glutaric acidemia (DYT/CHOR-GCDH)	#231670
- Methylmalonic aciduria (DYT/CHOR-MUT)	#251000
- Hartnup disease	#234500
- Ataxia telangiectasia	#208900
- Friedreich ataxia	#229300
- Neuroacanthocytosis	#200150
- Dopa-responsive dystonia (DYT/PARK-TH)	#605407
- Neuronal intranuclear hyaline inclusion disease	#603472
- Hereditary spastic paraplegia (HSP-SPG7)	#607259
- Sjögren–Larsson syndrome (ichthyosis, spasticity, intellectual disability)	#270200
- Biotin-responsive basal ganglia disease (DYT-SLC19A3)	#607483
- Dystonia musculorum deformans 2 (DYT-HPCA)	#224500
- Zech-boesch syndrom (DYT-31)	#619565
**X-linked recessive:**	
- Dystonia-parkinsonism or Lubag syndrome (DYT/PARK-TAF1)	#314250
- Lesch- Nyhan syndrome (DYT/CHOR-HPRT)	#300322
- Mohr-Tranebjaerg syndrome (Deafness–dystonia syndrome) (DYT-TIMM8A)	#304700
**X-linked dominant**	
- Rett syndrome	#312750
**Mitochondrial**	
- Leigh syndrome	#256000
- Leber’s hereditary ocular neuropathy plus dystonia (DYT-mt-ND6)	#500001

Legend: OMIM code = Online Mendelian Inheritance in Man code.

**Table 3:** Acquired form of dystonia.

**Perinatal Brain Injury:**
- Athetoid cerebral palsy - Delayed onset dystonia - Pachygyria
**Brain Injury:**
- Head trauma - Brain surgery (including stereotactic ablations, thalamotomy and thalamic lesions) - Lenticular nucleus lesions - Electrical injury - Cervical cord injury or lesion (including syringomyelia) - Peripheral injury - Lumbar canal stenosis
**Vascular:**
- Ischemia - Intracranial hemorrhage - Subdural hematoma arteriovenous malformation (including aneurysm); - Hypoxia
**Neoplastic and paraneoplastic:**
- Brain tumor (including posterior fossa tumor) - Paraneoplastic encephalitis (anti-Ma2-antibodies encephalopathy)
**Drug:**
- Levodopa and dopamine agonists - Dopamine D2 receptor-blocking drugs (Tardive dystonia; Acute dystonic reaction) - Anticonvulsants - Calcium channel blockers - Ergotism
**Toxic:**
- Manganese - Cobalt - Carbon disulphide - Cyanide - Methanol - Disulfiram - 3-nitropro-pionic acid - Wasp sting
**Metabolic:**
- Hypercalcemia - Hypoparathyroidism - Hypoglycemia - Hyperbilirubinemia - Pontine myelinolysis
**Encephalitis, infections, and post infections:**
- Reye syndrome - Poststreptococcal - Creutzfeldt–Jakob disease - Viral encephalitis - Encephalitis lethargica - Subacute sclerosing panencephalitis - Human immunodeficiency virus (HIV) infection - Other (tuberculosis, syphilis, cerebral abscess etc.)
**Autoimmune:**
- Primary antiphospholipid syndrome - Multiple sclerosis
**Immune encephalopathy:**
- Sjogren syndrome - Multiple myeloma - Rasmussen syndrome (NMDAR-antibodies), - Limbic encephalitic (LGI1-antibodies)
**Psychogenic (functional) dystonia**

## References

[1] Albanese A., Bhatia K., Bressman S.B., DeLong M.R., Fahn S., Fung V.S., Hallett M., Jankovic J., Jinnah H.A., Klein C. (2013). Phenomenology and classification of dystonia: A consensus update. Mov. Disord..

[2] Elia A., Lalli S., Albanese A. (2010). Differential diagnosis of dystonia. Eur. J. Neurol..

[3] Fahn S., Eldridge R. (1976). Definition of dystonia and classification of the dystonic states. Adv. Neurol..

[4] Fahn S., Marsden C.D., Calne D.B. (1987). Classification and investigation of dystonia. Mov. Disord..

[5] Fahn S., Bressman S.B., Marsden C.D. (1998). Classification of dystonia. Adv. Neurol..

[6] Albanese A., Asmus F., Bhatia K.P., Elia A.E., Elibol B., Filippini G., Gasser T., Krauss J.K., Nardocci N., Newton A. (2011). EFNS guidelines on diagnosis and treatment of primary dystonias. Eur. J. Neurol..

[7] Fahn S., Jankovic J., Hallett M. (2011). Principles and Practice of Movement Disorders.

[8] Albanese A., Jankovic J. (2012). Hyperkinetic Movement Disorders.

[9] Bhatia K.P., Bain P., Bajaj N., Elble R.J., Hallett M., Louis E.D., Raethjen J., Stamelou M., Testa C.M., Deuschl G. (2018). Consensus Statement on the classification of tremors. from the task force on tremor of the International Parkinson and Movement Disorder Society. Mov. Disord. Off. J. Mov. Disord. Soc..

[10] Schneider S.A., Edwards M.J., Mir P., Cordivari C., Hooker J., Dickson J., Quinn N., Bhatia K.P. (2007). Patients with adult-onset dystonic tremor resembling Parkinsonian tremor have scans without evidence of dopaminergic deficit (SWEDDs). Mov. Disord..

[11] Albanese A. (2003). The clinical expression of primary dystonia. J. Neurol..

[12] Vercueil L. (2006). Myoclonus and movement disorders. Neurophysiol. Clin..

[13] Gatto E.M., Pardal M.M.a.F., Micheli F.E. (2003). Unusual phenotypic expression of the DYT1 mutation. Parkinsonism Relat. Disord..

[14] Chinnery P.F., Reading P.J., McCarthy E.L., Curtis A., Burn D.J. (2002). Late-onset axial jerky dystonia due to the DYT1 deletion. Mov. Disord..

[15] Leung J., Klein C., Friedman J., Vieregge P., Jacobs H., Doheny D., Kamm C., DeLeon D., Pramstaller P.P., Penney J.B. (2001). Novel mutation in the TOR1A (DYT1) gene in atypical, early onset dystonia and polymorphisms in dystonia and early onset parkinsonism. Neurogenetics.

[16] Muller B., Hedrich K., Kock N., Dragasevic N., Svetel M., Garrels J., Landt O., Nitschke M., Pramstaller P.P., Reik W. (2002). Evidence that paternal expression of the epsilon-sarcoglycan gene accounts for reduced penetrance in myoclonus-dystonia. Am. J. Hum. Genet..

[17] Klein C. (2014). Genetics in dystonia. Parkinsonism Relat. Disord..

[18] Albanese A., Lalli S. (2009). Is this dystonia?. Mov. Disord..

[19] Cardoso F., Seppi K., Mair K.J., Wenning G.K., Poewe W. (2006). Seminar on choreas. Lancet Neurol..

[20] Fahn S., Hening W., Bressman S., Burke R., Ilson J., Walters A. (1985). Long-term usefulness of baclofen in the treatment of essential blepharospasm. Adv. Ophthalmic Plast. Reconstr. Surg..

[21] Cohen L.G., Hallett M. (1988). Hand cramps: Clinical features and electromyographic patterns in a focal dystonia. Neurology.

[22] Sitburana O., Wu L.J.C., Sheffield J.K., Davidson A., Jankovic J. (2009). Motor overflow and mirror dystonia. Parkinsonism Relat. Disord..

[23] Gömez-Wong E., Martí M.J., Cossu G., Fabregat N., Tolosa E.S., Valls-Solé J. (1998). The ‘geste antagonistique’ induces transient modulation of the blink reflex in human patients with blepharospasm. Neurosci. Lett..

[24] Greene P.E., Bressman S. (1998). Exteroceptive and interoceptive stimuli in dystonia. Mov. Disord..

[25] Hallett M. (1995). Is dystonia a sensory disorder?. Ann. Neurol..

[26] Jahanshahi M. (2000). Factors that ameliorate or aggravate spasmodic torticollis. J. Neurol. Neurosurg. Psychiatry.

[27] Müller J., Wissel J., Masuhr F., Ebersbach G., Wenning G.K., Poewe W. (2001). Clinical characteristics of the geste antagoniste in cervical dystonia. J. Neurol..

[28] Sanger T.D. (2003). Pathophysiology of pediatric movement disorders. J. Child Neurol..

[29] Barclay C.L., Lang A.E. (1997). Dystonia in progressive supranuclear palsy. J. Neurol. Neurosurg. Psychiatry.

[30] Vanek Z., Jankovic J. (2001). Dystonia in corticobasal degeneration. Mov. Disord. Off. J. Mov. Disord. Soc..

[31] Mastrolilli F., Benvenga A., Di Biase L., Giambattistelli F., Trotta L., Salomone G., Quintiliani L., Landi D., Melgari J., Vernieri F. (2011). An unusual cause of dementia: Essential diagnostic elements of corticobasal degeneration—A case report and review of the literature. Int. J. Alzheimer’s Dis..

[32] Herzog R., Weissbach A., Bäumer T., Münchau A. (2021). Complex dystonias: An update on diagnosis and care. J. Neural Transm..

[33] Rosencrantz R., Schilsky M. (2011). Wilson disease: Pathogenesis and clinical considerations in diagnosis and treatment. Semin. Liver Dis..

[34] Zech M., Jech R., Boesch S., Škorvánek M., Weber S., Wagner M., Zhao C., Jochim A., Necpál J., Dincer Y. (2020). Monogenic variants in dystonia: An exome-wide sequencing study. Lancet Neurol..

[35] Wirth T., Tranchant C., Drouot N., Keren B., Mignot C., Cif L., Lefaucheur R., Lion-François L., Méneret A., Gras D. (2020). Increased diagnostic yield in complex dystonia through exome sequencing. Parkinsonism Relat. Disord..

[36] Keller Sarmiento I.J., Mencacci N.E. (2021). Genetic Dystonias: Update on Classification and New Genetic Discoveries. Curr. Neurol. Neurosci. Rep..

[37] Vidailhet M., Méneret A., Roze E. (2020). Dystonia: Genetics, phenomenology, and pathophysiology. Lancet Neurol..

[38] Mencacci N.E., Reynolds R., Ruiz S.G., Vandrovcova J., Forabosco P., Sánchez-Ferrer A., Volpato V., Weale M.E., Bhatia K.P., Webber C. (2020). Dystonia genes functionally converge in specific neurons and share neurobiology with psychiatric disorders. Brain.

[39] Marras C., Lang A., van de Warrenburg B.P., Sue C.M., Tabrizi S.J., Bertram L., Mercimek-Mahmutoglu S., Ebrahimi-Fakhari D., Warner T.T., Durr A. (2016). Nomenclature of genetic movement disorders: Recommendations of the international Parkinson and movement disorder society task force. Mov. Disord. Off. J. Mov. Disord. Soc..

[40] Ozelius L.J., Hewett J.W., Page C.E., Bressman S.B., Kramer P.L., Shalish C., de Leon D., Brin M.F., Raymond D., Corey D.P. (1997). The early-onset torsion dystonia gene (DYT1) encodes an ATP-binding protein. Nat. Genet..

[41] Kabakci K., Hedrich K., Leung J.C., Mitterer M., Vieregge P., Lencer R., Hagenah J., Garrels J., Witt K., Klostermann F. (2004). Mutations in DYT1: Extension of the phenotypic and mutational spectrum. Neurology.

[42] Bressman S.B., Sabatti C., Raymond D., de Leon D., Klein C., Kramer P.L., Brin M.F., Fahn S., Breakefield X., Ozelius L.J. (2000). The DYT1 phenotype and guidelines for diagnostic testing. Neurology.

[43] Artusi C.A., Dwivedi A., Romagnolo A., Bortolani S., Marsili L., Imbalzano G., Sturchio A., Keeling E.G., Zibetti M., Contarino M.F. (2020). Differential response to pallidal deep brain stimulation among monogenic dystonias: Systematic review and meta-analysis. J. Neurol. Neurosurg. Psychiatry.

[44] Leube B., Rudnicki D., Ratzlaff T., Kessler K.R., Benecke R., Auburger G. (1996). Idiopathic torsion dystonia: Assignment of a gene to chromosome 18p in a German family with adult onset, autosomal dominant inheritance and purely focal distribution. Hum. Mol. Genet..

[45] Bressman S.B., Hunt A.L., Heiman G.A., Brin M.F., Burke R.E., Fahn S., Trugman J.M., de Leon D., Kramer P.L., Wilhelmsen K.C. (1994). Exclusion of the DYT1 locus in a non-Jewish family with early-onset dystonia. Mov. Disord. Off. J. Mov. Disord. Soc..

[46] Bentivoglio A.R., Del Grosso N., Albanese A., Cassetta E., Tonali P., Frontali M. (1997). Non-DYT1 dystonia in a large Italian family. J. Neurol. Neurosurg. Psychiatry.

[47] Camargos S., Scholz S., Simon-Sanchez J., Paisan-Ruiz C., Lewis P., Hernandez D., Ding J., Gibbs J.R., Cookson M.R., Bras J. (2008). DYT16, a novel young-onset dystonia-parkinsonism disorder: Identification of a segregating mutation in the stress-response protein PRKRA. Lancet Neurol..

[48] Seibler P., Djarmati A., Langpap B., Hagenah J., Schmidt A., Bruggemann N., Siebner H., Jabusch H.C., Altenmuller E., Munchau A. (2008). A heterozygous frameshift mutation in PRKRA (DYT16) associated with generalised dystonia in a German patient. Lancet Neurol..

[49] Klein C., Lohmann K., Marras C., Münchau A., Adam M.P., Ardinger H.H., Pagon R.A., Wallace S.E., Bean L.J.H., Stephens K., Amemiya A. (1993). Hereditary Dystonia Overview. GeneReviews^®^.

[50] Klein C., Fahn S. (2013). Translation of Oppenheim’s 1911 paper on dystonia. Mov. Disord. Off. J. Mov. Disord. Soc..

[51] Tadic V., Kasten M., Bruggemann N., Stiller S., Hagenah J., Klein C. (2012). Dopa-responsive dystonia revisited: Diagnostic delay, residual signs, and nonmotor signs. Arch. Neurol..

[52] Brashear A., Dobyns W.B., de Carvalho Aguiar P., Borg M., Frijns C.J., Gollamudi S., Green A., Guimaraes J., Haake B.C., Klein C. (2007). The phenotypic spectrum of rapid-onset dystonia-parkinsonism (RDP) and mutations in the ATP1A3 gene. Brain.

[53] Rughani A.I., Lozano A.M. (2013). Surgical treatment of myoclonus dystonia syndrome. Mov. Disord. Off. J. Mov. Disord. Soc..

[54] Mencacci N.E., Rubio-Agusti I., Zdebik A., Asmus F., Ludtmann M.H., Ryten M., Plagnol V., Hauser A.K., Bandres-Ciga S., Bettencourt C. (2015). A missense mutation in KCTD17 causes autosomal dominant myoclonus-dystonia. Am. J. Hum. Genet..

[55] Todisco M., Gana S., Cosentino G., Errichiello E., Arceri S., Avenali M., Valente E.M., Alfonsi E. (2020). KCTD17-related myoclonus-dystonia syndrome: Clinical and electrophysiological findings of a patient with atypical late onset. Parkinsonism Relat. Disord..

[56] Chinnery P.F., Adam M.P., Ardinger H.H., Pagon R.A., Wallace S.E., Bean L.J.H., Stephens K., Amemiya A. (1993). Neuroferritinopathy. GeneReviews^®^.

[57] Van de Warrenburg B.P., van Gaalen J., Boesch S., Burgunder J.M., Dürr A., Giunti P., Klockgether T., Mariotti C., Pandolfo M., Riess O. (2014). EFNS/ENS Consensus on the diagnosis and management of chronic ataxias in adulthood. Eur. J. Neurol..

[58] Iizuka R., Hirayama K., Maehara K.A. (1984). Dentato-rubro-pallido-luysian atrophy: A clinico-pathological study. J. Neurol. Neurosurg. Psychiatry.

[59] Hatano T., Okuma Y., Iijima M., Fujishima K., Goto K., Mizuno Y. (2003). Cervical dystonia in dentatorubral-pallidoluysian atrophy. Acta Neurol. Scand..

[60] Richards R.I. (2001). Dynamic mutations: A decade of unstable expanded repeats in human genetic disease. Hum. Mol. Genet..

[61] Becker N., Munhoz R.P., Raskin S., Werneck L.C., Teive H.A. (2007). Non-choreic movement disorders as initial manifestations of Huntington’s disease. Arq. Neuro-Psiquiatr..

[62] Van de Zande N.A., Massey T.H., McLauchlan D., Pryce Roberts A., Zutt R., Wardle M., Payne G.C., Clenaghan C., Tijssen M.A.J., Rosser A.E. (2017). Clinical characterization of dystonia in adult patients with Huntington’s disease. Eur. J. Neurol..

[63] De Castilhos R.M., Furtado G.V., Gheno T.C., Schaeffer P., Russo A., Barsottini O., Pedroso J.L., Salarini D.Z., Vargas F.R., de Lima M.A. (2014). Spinocerebellar ataxias in Brazil--frequencies and modulating effects of related genes. Cerebellum.

[64] Jardim L.B., Pereira M.L., Silveira I., Ferro A., Sequeiros J., Giugliani R. (2001). Neurologic findings in Machado-Joseph disease: Relation with disease duration, subtypes, and (CAG)n. Arch. Neurol..

[65] Nunes M.B., Martinez A.R., Rezende T.J., Friedman J.H., Lopes-Cendes I., D’Abreu A., França M.C. (2015). Dystonia in Machado-Joseph disease: Clinical profile, therapy and anatomical basis. Parkinsonism Relat. Disord..

[66] Masters C.L., Richardson E.P. (1978). Subacute spongiform encephalopathy (Creutzfeldt-Jakob disease). The nature and progression of spongiform change. Brain.

[67] Kaneko A., Takei K., Enomoto K., Mitsui T., Nomura K., Iwasaki S., Maruki T., Shimazu K. (1999). A case of Creutzfeldt-Jakob disease exhibiting athetosis in the early stage. No Shinkei.

[68] Maltete D., Guyant-Marechal L., Gerardin E., Laquerriere A., Martinaud O., Mihout B., Hannequin D. (2006). Hemidystonia as initial manifestation of sporadic Creutzfeldt-Jakob disease. Eur. J. Neurol..

[69] Sanchez-Contreras M., Baker M.C., Finch N.A., Nicholson A., Wojtas A., Wszolek Z.K., Ross O.A., Dickson D.W., Rademakers R. (2014). Genetic screening and functional characterization of PDGFRB mutations associated with basal ganglia calcification of unknown etiology. Hum. Mutat..

[70] Legati A., Giovannini D., Nicolas G., López-Sánchez U., Quintáns B., Oliveira J.R., Sears R.L., Ramos E.M., Spiteri E., Sobrido M.J. (2015). Mutations in XPR1 cause primary familial brain calcification associated with altered phosphate export. Nat. Genet..

[71] Lange L.M., Junker J., Loens S., Baumann H., Olschewski L., Schaake S., Madoev H., Petkovic S., Kuhnke N., Kasten M. (2021). Genotype-Phenotype Relations for Isolated Dystonia Genes: MDSGene Systematic Review. Mov. Disord. Off. J. Mov. Disord. Soc..

[72] Steel D., Zech M., Zhao C., Barwick K.E.S., Burke D., Demailly D., Kumar K.R., Zorzi G., Nardocci N., Kaiyrzhanov R. (2020). Loss-of-Function Variants in HOPS Complex Genes VPS16 and VPS41 Cause Early Onset Dystonia Associated with Lysosomal Abnormalities. Ann. Neurol..

[73] Monfrini E., Zech M., Steel D., Kurian M.A., Winkelmann J., Di Fonzo A. (2021). HOPS-associated neurological disorders (HOPSANDs): Linking endolysosomal dysfunction to the pathogenesis of dystonia. Brain.

[74] Kuipers D.J.S., Mandemakers W., Lu C.S., Olgiati S., Breedveld G.J., Fevga C., Tadic V., Carecchio M., Osterman B., Sagi-Dain L. (2021). EIF2AK2 Missense Variants Associated with Early Onset Generalized Dystonia. Ann. Neurol..

[75] Magrinelli F., Moualek D., Tazir M., Ali Pacha L., Verghese A., Bhatia K.P., Maroofian R., Houlden H. (2021). Heterozygous EIF2AK2 variant causes adolescence-onset generalized dystonia partially responsive to DBS. Mov. Disord. Clin. Pract..

[76] Ebrahimi-Fakhari D., Van Karnebeek C., Munchau A. (2019). Movement Disorders in Treatable Inborn Errors of Metabolism. Mov. Disord. Off. J. Mov. Disord. Soc..

[77] Svetel M., Kozic D., Stefanova E., Semnic R., Dragasevic N., Kostic V.S. (2001). Dystonia in Wilson’s disease. Mov. Disord. Off. J. Mov. Disord. Soc..

[78] Gregory A., Hayflick S., Adam M.P., Ardinger H.H., Pagon R.A., Wallace S.E., Bean L.J.H., Stephens K., Amemiya A. (2019). Neurodegeneration with Brain Iron Accumulation Disorders Overview. GeneReviews^®^.

[79] Miyajima H., Hosoi Y., Adam M.P., Ardinger H.H., Pagon R.A., Wallace S.E., Bean L.J.H., Stephens K., Amemiya A. (1993). Aceruloplasminemia. GeneReviews^®^.

[80] Ahlskog J.E. (2009). Parkin and PINK1 parkinsonism may represent nigral mitochondrial cytopathies distinct from Lewy body Parkinson’s disease. Parkinsonism Relat. Disord..

[81] Valente E.M., Abou-Sleiman P.M., Caputo V., Muqit M.M., Harvey K., Gispert S., Ali Z., Del Turco D., Bentivoglio A.R., Healy D.G. (2004). Hereditary early-onset Parkinson’s disease caused by mutations in PINK1. Science.

[82] Brüggemann N., Spiegler J., Hellenbroich Y., Opladen T., Schneider S.A., Stephani U., Boor R., Gillessen-Kaesbach G., Sperner J., Klein C. (2012). Beneficial prenatal levodopa therapy in autosomal recessive guanosine triphosphate cyclohydrolase 1 deficiency. Arch. Neurol..

[83] Himmelreich N., Montioli R., Bertoldi M., Carducci C., Leuzzi V., Gemperle C., Berner T., Hyland K., Thöny B., Hoffmann G.F. (2019). Aromatic amino acid decarboxylase deficiency: Molecular and metabolic basis and therapeutic outlook. Mol. Genet. Metab..

[84] Vanier M.T. (2010). Niemann-Pick disease type C. Orphanet J. Rare Dis..

[85] Koens L.H., Kuiper A., Coenen M.A., Elting J.W., de Vries J.J., Engelen M., Koelman J.H., van Spronsen F.J., Spikman J.M., de Koning T.J. (2016). Ataxia, dystonia and myoclonus in adult patients with Niemann-Pick type C. Orphanet J. Rare Dis..

[86] Mitchison H.M., Hofmann S.L., Becerra C.H., Munroe P.B., Lake B.D., Crow Y.J., Stephenson J.B., Williams R.E., Hofman I.L., Taschner P.E. (1998). Mutations in the palmitoyl-protein thioesterase gene (PPT.; CLN1) causing juvenile neuronal ceroid lipofuscinosis with granular osmiophilic deposits. Hum. Mol. Genet..

[87] Yıldırım M., Köse E., Keçeli A.M., Balasar Ö., Şimşek N. (2020). Status dystonicus associated with CLN8 disease. Brain Dev..

[88] Arash-Kaps L., Komlosi K., Seegräber M., Diederich S., Paschke E., Amraoui Y., Beblo S., Dieckmann A., Smitka M., Hennermann J.B. (2019). The Clinical and Molecular Spectrum of GM1 Gangliosidosis. J. Pediatr..

[89] Meek D., Wolfe L.S., Andermann E., Andermann F. (1984). Juvenile progressive dystonia: A new phenotype of GM2 gangliosidosis. Ann. Neurol..

[90] van Rappard D.F., Boelens J.J., Wolf N.I. (2015). Metachromatic leukodystrophy: Disease spectrum and approaches for treatment. Best Prac. Res. Clin. Endocrinol. Metab..

[91] Carson N.A., Dent C.E., Field C.M., Gaull G.E. (1965). Homocystinuria: Clinical and pathological review of ten cases. J. Pediatr..

[92] Biery B.J., Stein D.E., Morton D.H., Goodman S.I. (1996). Gene structure and mutations of glutaryl-coenzyme A dehydrogenase: Impaired association of enzyme subunits that is due to an A421V substitution causes glutaric acidemia type I in the Amish. Am. J. Hum. Genet..

[93] Kölker S., Burgard P., Sauer S.W., Okun J.G. (2013). Current concepts in organic acidurias: Understanding intra- and extracerebral disease manifestation. J. Inherit. Metab. Dis..

[94] Cossée M., Dürr A., Schmitt M., Dahl N., Trouillas P., Allinson P., Kostrzewa M., Nivelon-Chevallier A., Gustavson K.H., Kohlschütter A. (1999). Friedreich’s ataxia: Point mutations and clinical presentation of compound heterozygotes. Ann. Neurol..

[95] Davidson C.S., Stanbury J.B., Wyngaarden B.J., Fredrickson D.S., Goldstein J.L., Brown M.S. (1983). The Metabolic Basis of Inherited Disease.

[96] Darras B.T., Ampola M.G., Dietz W.H., Gilmore H.E. (1989). Intermittent dystonia in Hartnup disease. Pediatric Neurol..

[97] Kim M., Kim A.R., Park J., Kim J.S., Ahn J.H., Park W.Y., Kim N.K.D., Lee C., Kim N.S., Cho J.W. (2020). Clinical characteristics of ataxia-telangiectasia presenting dystonia as a main manifestation. Clin. Neurol. Neurosurg..

[98] Van Os N.J.H., Hensiek A., van Gaalen J., Taylor A.M.R., van Deuren M., Weemaes C.M.R., Willemsen M., van de Warrenburg B.P.C. (2019). Trajectories of motor abnormalities in milder phenotypes of ataxia telangiectasia. Neurology.

[99] Levy A., Lang A.E. (2018). Ataxia-telangiectasia: A review of movement disorders, clinical features, and genotype correlations. Mov. Disord. Off. J. Mov. Disord. Soc..

[100] Campuzano V., Montermini L., Moltò M.D., Pianese L., Cossée M., Cavalcanti F., Monros E., Rodius F., Duclos F., Monticelli A. (1996). Friedreich’s ataxia: Autosomal recessive disease caused by an intronic GAA triplet repeat expansion. Science.

[101] Rampoldi L., Danek A., Monaco A.P. (2002). Clinical features and molecular bases of neuroacanthocytosis. J. Mol. Med..

[102] Danek A., Jung H.H., Melone M.A., Rampoldi L., Broccoli V., Walker R.H. (2005). Neuroacanthocytosis: New developments in a neglected group of dementing disorders. J. Neurol. Sci..

[103] Paviour D.C., Revesz T., Holton J.L., Evans A., Olsson J.E., Lees A.J. (2005). Neuronal intranuclear inclusion disease: Report on a case originally diagnosed as dopa-responsive dystonia with Lewy bodies. Mov. Disord. Off. J. Mov. Disord. Soc..

[104] Kish S.J., Gilbert J.J., Chang L.J., Mirchandani L., Shannak K., Hornykiewicz O. (1985). Brain neurotransmitter abnormalities in neuronal intranuclear inclusion body disorder. Ann. Neurol..

[105] Goutières F., Mikol J., Aicardi J. (1990). Neuronal intranuclear inclusion disease in a child: Diagnosis by rectal biopsy. Ann. Neurol..

[106] Garen P.D., Powers J.M., Young G.F., Lee V. (1986). Neuronal intranuclear hyaline inclusion disease in a nine year old. Acta Neuropathol..

[107] Schaefer S.M., Szekely A.M., Moeller J.J., Tinaz S. (2018). Hereditary spastic paraplegia presenting as limb dystonia with a rare SPG7 mutation. Neurol. Clin. Pract..

[108] Pfeffer G., Gorman G.S., Griffin H., Kurzawa-Akanbi M., Blakely E.L., Wilson I., Sitarz K., Moore D., Murphy J.L., Alston C.L. (2014). Mutations in the SPG7 gene cause chronic progressive external ophthalmoplegia through disordered mitochondrial DNA maintenance. Brain.

[109] Daoud H., Papadima E.M., Ouled Amar Bencheikh B., Katsila T., Dionne-Laporte A., Spiegelman D., Dion P.A., Patrinos G.P., Orrù S., Rouleau G.A. (2015). Identification of a novel homozygous SPG7 mutation by whole exome sequencing in a Greek family with a complicated form of hereditary spastic paraplegia. Eur. J. Med. Genet..

[110] Marcotulli C., Leonardi L., Tessa A., De Negris A.M., Cornia R., Pierallini A., Haggiag S., Pierelli F., Santorelli F.M., Casali C. (2014). Early-onset optic neuropathy as initial clinical presentation in SPG7. J. Neurol..

[111] Roxburgh R.H., Marquis-Nicholson R., Ashton F., George A.M., Lea R.A., Eccles D., Mossman S., Bird T., van Gassen K.L., Kamsteeg E.J. (2013). The p.Ala510Val mutation in the SPG7 (paraplegin) gene is the most common mutation causing adult onset neurogenetic disease in patients of British ancestry. J. Neurol..

[112] Van Gassen K.L., van der Heijden C.D., de Bot S.T., den Dunnen W.F., van den Berg L.H., Verschuuren-Bemelmans C.C., Kremer H.P., Veldink J.H., Kamsteeg E.J., Scheffer H. (2012). Genotype-phenotype correlations in spastic paraplegia type 7: A study in a large Dutch cohort. Brain.

[113] Sjogren T., Larsson T. (1957). Oligophrenia in combination with congenital ichthyosis and spastic disorders; a clinical and genetic study. Acta Psychiatr. Neurol. Scand. Suppl..

[114] Cubo E., Goetz C.G. (2000). Dystonia secondary to Sjogren-Larsson syndrome. Neurology.

[115] Zeng W.Q., Al-Yamani E., Acierno J.S., Slaugenhaupt S., Gillis T., MacDonald M.E., Ozand P.T., Gusella J.F. (2005). Biotin-responsive basal ganglia disease maps to 2q36.3 and is due to mutations in SLC19A3. Am. J. Hum. Genet..

[116] Charlesworth G., Angelova P.R., Bartolomé-Robledo F., Ryten M., Trabzuni D., Stamelou M., Abramov A.Y., Bhatia K.P., Wood N.W. (2015). Mutations in HPCA cause autosomal-recessive primary isolated dystonia. Am. J. Hum. Genet..

[117] Zech M., Kumar K.R., Reining S., Reunert J., Tchan M., Riley L.G., Drew A.P., Adam R.J., Berutti R., Biskup S. (2021). Biallelic AOPEP Loss-of-Function Variants Cause Progressive Dystonia with Prominent Limb Involvement. Mov. Disord. Off. J. Mov. Disord. Soc..

[118] Lee L.V., Rivera C., Teleg R.A., Dantes M.B., Pasco P.M., Jamora R.D., Arancillo J., Villareal-Jordan R.F., Rosales R.L., Demaisip C. (2011). The unique phenomenology of sex-linked dystonia parkinsonism (XDP, DYT3, “Lubag”). Int. J. Neurosci..

[119] Balint B., Mencacci N.E., Valente E.M., Pisani A., Rothwell J., Jankovic J., Vidailhet M., Bhatia K.P. (2018). Dystonia. Nat. Rev. Dis. Primers.

[120] Nanagiri A., Shabbir N. (2020). Lesch Nyhan Syndrome. StatPearls.

[121] Fu R., Sutcliffe D., Zhao H., Huang X., Schretlen D.J., Benkovic S., Jinnah H.A. (2015). Clinical severity in Lesch-Nyhan disease: The role of residual enzyme and compensatory pathways. Mol. Genet. Metab..

[122] Jin H., May M., Tranebjaerg L., Kendall E., Fontan G., Jackson J., Subramony S.H., Arena F., Lubs H., Smith S. (1996). A novel X-linked gene, DDP, shows mutations in families with deafness (DFN-1), dystonia, mental deficiency and blindness. Nat. Genet..

[123] Ha A.D., Parratt K.L., Rendtorff N.D., Lodahl M., Ng K., Rowe D.B., Sue C.M., Hayes M.W., Tranebjaerg L., Fung V.S. (2012). The phenotypic spectrum of dystonia in Mohr-Tranebjaerg syndrome. Mov. Disord. Off. J. Mov. Disord. Soc..

[124] Amir R.E., Van den Veyver I.B., Wan M., Tran C.Q., Francke U., Zoghbi H.Y. (1999). Rett syndrome is caused by mutations in X-linked MECP2, encoding methyl-CpG-binding protein 2. Nat. Genet..

[125] Lake N.J., Compton A.G., Rahman S., Thorburn D.R. (2016). Leigh syndrome: One disorder, more than 75 monogenic causes. Ann. Neurol..

[126] Tranchant C., Anheim M. (2016). Movement disorders in mitochondrial diseases. Rev. Neurol..

[127] Yu-Wai-Man P., Turnbull D.M., Chinnery P.F. (2002). Leber hereditary optic neuropathy. J. Med. Genet..

[128] Nikoskelainen E.K., Marttila R.J., Huoponen K., Juvonen V., Lamminen T., Sonninen P., Savontaus M.L. (1995). Leber’s “plus”: Neurological abnormalities in patients with Leber’s hereditary optic neuropathy. J. Neurol. Neurosurg. Psychiatry.

[129] Dressler D. (2011). Nonprimary dystonias. Handb. Clin. Neurol..

[130] Michael-Asalu A., Taylor G., Campbell H., Lelea L.L., Kirby R.S. (2019). Cerebral Palsy: Diagnosis, Epidemiology, Genetics, and Clinical Update. Adv. Pediatrics.

[131] Chuang C., Fahn S., Frucht S.J. (2002). The natural history and treatment of acquired hemidystonia: Report of 33 cases and review of the literature. J. Neurol. Neurosurg. Psychiatry.

[132] Cammarota A., Gershanik O.S., Garcia S., Lera G. (1995). Cervical dystonia due to spinal cord ependymoma: Involvement of cervical cord segments in the pathogenesis of dystonia. Mov. Disord. Off. J. Mov. Disord. Soc..

[133] Jinnah H.A. (2019). The Dystonias. Continuum.

[134] De Keyser J. (1993). Subtypes and localization of dopamine receptors in human brain. Neurochem. Int..

[135] Marano M., di Biase L., Salomone G., Di Santo A., Montiroli A., Di Lazzaro V. (2016). The Clinical Course of a Drug-induced Acute Dystonic Reaction in the Emergency Room. Tremor Other Hyperkinetic Mov..

[136] Choi I.S., Cheon H.Y. (1999). Delayed movement disorders after carbon monoxide poisoning. Eur. Neurol..

[137] Moulick A., Sengupta P., Banerjee S., Basu D. (2008). Oromandibular dystonia and persistent psychiatric symptoms in extra-pontine myelinolysis. J. Assoc. Physicians India.

[138] Shah I., Chudgar P. (2005). Progressive multifocal leukoencephalopathy (PML) presenting as intractable dystonia in an HIV-infected child. J. Trop. Pediatrics.

[139] Angelini L., Rumi V., Nardocci N., Combi M.L., Bruzzone M.G., Pellegrini G. (1993). Hemidystonia symptomatic of primary antiphospholipid syndrome in childhood. Mov. Disord. Off. J. Mov. Disord. Soc..

[140] Fahn S., Williams D.T. (1988). Psychogenic dystonia. Adv. Neurol..

[141] Oppenheim H. (1911). About a rare spasm disease of childhood and young age (Dysbasia lordotica progressiva, dystonia musculorum deformans). Neurol. Cent..

[142] Schramm A., Classen J., Reiners K., Naumann M. (2007). Characteristics of sensory trick-like manoeuvres in jaw-opening dystonia. Mov. Disord..

[143] Dressler D. (2010). Botulinum toxin for treatment of dystonia. Eur. J. Neurol..

[144] Engstrom P.F., Arnoult J.B., Mazow M.L., Prager T.C., Wilkins R.B., Byrd W.A., Hofmann R.J. (1987). Effectiveness of botulinum toxin therapy for essential blepharospasm. Ophthalmology.

[145] Albanese A., Colosimo C., Carretta D., Dickmann A., Bentivoglio A.R., Tonali P. (1992). Botulinum toxin as a treatment for blepharospasm, spasmodic torticollis and hemifacial spasm. Eur. Neurol..

[146] Ababneh O.H., Cetinkaya A., Kulwin D.R. (2014). Long-term efficacy and safety of botulinum toxin A injections to treat blepharospasm and hemifacial spasm. Clin. Exp. Ophthalmol..

[147] Kessler K.R., Skutta M., Benecke R., Group G.D.S. (1999). Long-term treatment of cervical dystonia with botulinum toxin A: Efficacy, safety, and antibody frequency. J. Neurol..

[148] Behari M. (1999). Botulinum toxin in the treatment of writer’s cramp. J. Assoc. Physicians India.

[149] Turjanski N., Pirtosek Z., Quirk J., Anderson T., Rivest J., Marsden C., Lees A. (1996). Botulinum toxin in the treatment of writer’s cramp. Clin. Neuropharmacol..

[150] Schuele S., Jabusch H.-C., Lederman R.J., Altenmüller E. (2005). Botulinum toxin injections in the treatment of musician’s dystonia. Neurology.

[151] Yoshimura D.M., Aminoff M.J., Olney R.K. (1992). Botulinum toxin therapy for limb dystonias. Neurology.

[152] Pullman S.L., Greene P., Fahn S., Pedersen S.F. (1996). Approach to the treatment of limb disorders with botulinum toxin A: Experience with 187 patients. Arch. Neurol..

[153] Quirk J., Sheean G., Marsden C., Lees A. (1996). Treatment of nonoccupational limb and trunk dystonia with botulinum toxin. Mov. Disord. Off. J. Mov. Disord. Soc..

[154] Bonanni L., Thomas A., Varanese S., Scorrano V., Onofrj M. (2007). Botulinum toxin treatment of lateral axial dystonia in Parkinsonism. Mov. Disord. Off. J. Mov. Disord. Soc..

[155] di Biase L., Fasano A. (2016). Low-frequency deep brain stimulation for Parkinson’s disease: Great expectation or false hope?. Mov. Disord..

[156] Kern D.S., Picillo M., Thompson J.A., Sammartino F., di Biase L., Munhoz R.P., Fasano A. (2018). Interleaving Stimulation in Parkinson’s Disease, Tremor, and Dystonia. Stereotact. Funct. Neurosurg..

[157] Benabid A.L., Chabardes S., Mitrofanis J., Pollak P. (2009). Deep brain stimulation of the subthalamic nucleus for the treatment of Parkinson’s disease. Lancet Neurol..

[158] Krack P., Batir A., Van Blercom N., Chabardes S., Fraix V., Ardouin C., Koudsie A., Limousin P.D., Benazzouz A., LeBas J.F. (2003). Five-year follow-up of bilateral stimulation of the subthalamic nucleus in advanced Parkinson’s disease. N. Engl. J. Med..

[159] Sandoe C., Krishna V., Basha D., Sammartino F., Tatsch J., Picillo M., di Biase L., Poon Y.Y., Hamani C., Reddy D. (2018). Predictors of deep brain stimulation outcome in tremor patients. Brain Stimul..

[160] Benabid A.L., Pollak P., Gervason C., Hoffmann D., Gao D.M., Hommel M., Perret J.E., de Rougemont J. (1991). Long-term suppression of tremor by chronic stimulation of the ventral intermediate thalamic nucleus. Lancet.

[161] Koller W.C., Lyons K.E., Wilkinson S.B., Troster A.I., Pahwa R. (2001). Long-term safety and efficacy of unilateral deep brain stimulation of the thalamus in essential tremor. Mov. Disord..

[162] Di Biase L., Munhoz R.P. (2016). Deep brain stimulation for the treatment of hyperkinetic movement disorders. Expert Rev. Neurother..

[163] Mehrkens J.H., Botzel K., Steude U., Zeitler K., Schnitzler A., Sturm V., Voges J. (2009). Long-term efficacy and safety of chronic globus pallidus internus stimulation in different types of primary dystonia. Stereotact. Funct. Neurosurg..

[164] Cif L., El Fertit H., Vayssiere N., Hemm S. (2003). Treatment of dystonic syndromes by chronic electrical stimulation of the internal globus pallidus. J. Neurosurg. Sci..

[165] Kupsch A., Benecke R., Muller J., Trottenberg T., Schneider G.H., Poewe W., Eisner W., Wolters A., Muller J.U., Deuschl G. (2006). Pallidal deep-brain stimulation in primary generalized or segmental dystonia. N. Engl. J. Med..

[166] Vidailhet M., Vercueil L., Houeto J.L., Krystkowiak P., Benabid A.L., Cornu P., Lagrange C., Du Montcel S.T., Dormont D., Grand S. (2005). Bilateral deep-brain stimulation of the globus pallidus in primary generalized dystonia. N. Engl. J. Med..

[167] Giovanni A., Capone F., di Biase L., Ferreri F., Florio L., Guerra A., Marano M., Paolucci M., Ranieri F., Salomone G. (2017). Oscillatory Activities in Neurological Disorders of Elderly: Biomarkers to Target for Neuromodulation. Front. Aging Neurosci..

[168] Assenza G., Capone F., di Biase L., Ferreri F., Florio L., Guerra A., Marano M., Paolucci M., Ranieri F., Salomone G. (2017). Corrigendum: Oscillatory Activities in Neurological Disorders of Elderly: Biomarkers to Target for Neuromodulation. Front. Aging Neurosci..

[169] Little S., Pogosyan A., Neal S., Zavala B., Zrinzo L., Hariz M., Foltynie T., Limousin P., Ashkan K., FitzGerald J. (2013). Adaptive deep brain stimulation in advanced Parkinson disease. Ann. Neurol..

[170] Little S., Pogosyan A., Neal S., Zrinzo L., Hariz M., Foltynie T., Limousin P., Brown P. (2014). Controlling Parkinson’s disease with adaptive deep brain stimulation. JoVE.

[171] Tinkhauser G., Pogosyan A., Debove I., Nowacki A., Shah S.A., Seidel K., Tan H., Brittain J.S., Petermann K., di Biase L. (2018). Directional local field potentials: A tool to optimize deep brain stimulation. Mov. Disord. Off. J. Mov. Disord. Soc..

[172] Pina-Fuentes D., Beudel M., Little S., van Zijl J., Elting J.W., Oterdoom D.L.M., van Egmond M.E., van Dijk J.M.C., Tijssen M.A.J. (2018). Toward adaptive deep brain stimulation for dystonia. Neurosurg. Focus.

